# Associations between birthweight and preterm birth and the ages at menarche and menopause

**DOI:** 10.1186/s12905-024-03384-6

**Published:** 2024-10-03

**Authors:** Christian Daniele, Rachel E. Wacks, Leslie V. Farland, JoAnn E. Manson, Lihong Qi, Aladdin H. Shadyab, Sylvia Wassertheil-Smoller, Cassandra N. Spracklen

**Affiliations:** 1 Department of Biostatistics and Epidemiology, University of Massachusetts-Amherst, 715 North Pleasant Street, Amherst, MA 01003 USA; 2 Department of Epidemiology and Biostatistics, Mel and Enid Zuckerman College of Public Health, University of Arizona, 1295 N. Martin Ave., Tucson, AZ 85724 USA; 3 Department of Epidemiology, Harvard T.H. Chan School of Public Health, Boston, MA USA; 4 Division of Preventive Medicine, Department of Medicine, Brigham and Women’s Hospital and Harvard Medical School, 900 Commonwealth Ave., Boston, MA 02215 USA; 5 Department of Public Health Sciences, The University of California Davis, One Shields Ave., Med-Sci 1C Room 145, Davis, CA 95616 USA; 6 Herbert Wertheim School of Public Health and Human Longevity Science, University of California, 9500 Gilman Drive #0725, San Diego, La Jolla, CA 92093 USA; 7 Department of Epidemiology and Population Health, Albert Einstein College of Medicine, Bronx, NY 10461 USA

**Keywords:** Birthweight, Preterm birth, Women’s health, Menarche, Menopause

## Abstract

**Background:**

Women who reach menarche and menopause at earlier ages have been shown to be at increased risk for numerous conditions including cardiovascular disease, cancer, depression, and obesity; however, risk factors for earlier ages of menarche and menopause are not fully understood. Therefore, we aimed to perform a retrospective investigation of the associations between a personal birthweight and/or being born preterm and the age of and menarche and menopause and related events in the Women’s Health Initiative, a large, racially and ethnically diverse cohort of postmenopausal women.

**Methods:**

At study entry, women reported their birthweight by category (< 6 lbs., 6–7 lbs. 15 oz, 8–9 lbs. 15 oz, or ≥ 10 lbs.) and preterm birth status (4 or more weeks premature). Ages at events related to menarche and menopause were also self-reported. Linear regression and logistic regression models were used to estimate unadjusted and adjusted effect estimates (β) and odds ratios (OR), respectively (*n* ≤ 86,857). Individuals born preterm were excluded from all birthweight analyses.

**Results:**

After adjustments, individuals born weighing < 6lbs. were more likely to reach natural menopause at an earlier age (adjusted β=-0.361, SE = 0.09, P = < 0.001) and have a shorter reproductive window (adjusted β = -0.287, SE = 0.10, *p* < 0.004) compared to individuals weighing 6–7 lbs. 15 oz. Individuals born preterm were also more likely to reach natural menopause at an earlier age (adjusted β=-0.506, SE = 0.16, *P* = 0.001) and have a shorter reproductive window (adjusted β = -0.418, SE = 0.17, *p* < 0.006).

**Conclusions:**

These findings raise concerns that, as more preterm and low birthweight individuals survive to adulthood, the prevalence of earlier-onset menarche and menopause may increase. Clinical counseling and interventions aimed at reducing the incidence of preterm and low birthweight births, as well as intensification of lifestyle modifications to reduce CVD risk among women with these early-life risk factors, should be prioritized.

**Supplementary Information:**

The online version contains supplementary material available at 10.1186/s12905-024-03384-6.

## Introduction

A woman’s age at menarche and menopause marks the start and end of her reproductive years, respectively. The timing of these hormonal transition periods is critical for a woman’s health trajectory over her lifespan, as they are indicators of ovarian function and aging. A growing body of literature consistently shows that both early and late onset of either event is associated with increased risk for adverse health outcomes. An earlier age of menarche has demonstrated associations with increased risk of breast and endometrial cancers, depression, cardiovascular disease, obesity, and premature death, while later age of menarche is associated with higher risk of depression and lower bone mineral density [1]. Earlier age at menopause has been associated with higher risk of cardiovascular disease, lower bone mineral density, osteoporosis, and premature death; later timing of menopause puts a woman at increased risk for breast and endometrial cancers [1]. Therefore, identifying risk factors for early- and/or late-onset of menarche and menopause might have implications for public health, chronic disease prevention, and improvement in quality of life.

The Developmental Origins of Health and Disease hypothesis, or “Barker hypothesis”, postulates that diseases occurring across the lifecourse result from environmental exposures *in utero* and early childhood [2]. The hypothesis has been frequently supported within chronic conditions that affect individuals later in life, such as cardiovascular disease, cancer, disability, thyroid conditions, autoimmune diseases, and type 2 diabetes [3–7]. However, the association of early life exposures, such as birthweight and/or preterm birth, on the development of women’s health-related outcomes, including age at menarche and menopause, has been less well studied. At least 13 studies have examined the association between a woman’s own birthweight and age at menarche, suggesting those born at higher birthweights reach menarche at a later age [8]. Fewer studies have examined the association between a woman’s birthweight and her age at natural menopause, suggesting either no association or that those born at higher birthweights reach menopause at a later age [9–13]. Additionally, the literature examining the timing of menarche and menopause in women born preterm is limited [12, 14–16]. Overall, most of the prior studies were conducted in small samples (*N* < 1,000) and/or the study populations were composed of predominantly White individuals from Europe or Australia. Therefore, the goal of our study was to investigate the association between an individual’s birthweight and gestational age at delivery and ages at menarche and menopause and related events among participants in the Women’s Health Initiative (WHI).

## Methods

### Study population

An ongoing prospective cohort study, the Women’s Health Initiative (WHI), was designed to examine socio-demographic, behavioral, and clinical risk factors for several disease outcomes in post-menopausal women. At enrollment, 161,608 post-menopausal women between ages 50 and 79 in the United States were recruited from the general population at 40 clinical recruitment sites between 1993 and 1998 [17, 18]. Participants were given the opportunity to enroll into overlapping clinical trials (WHI-CT; *n* = 67,932) or a long-term observational study (WHI-OS; *n* = 93,676). Further details on study design, recruitment, and implementation have been described elsewhere [17]. The Institutional Review Board of each precipitating recruitment site approved all study protocols, and all participants provided written, informed consent at study commencement.

### Baseline measures

Upon enrollment, women in the WHI-OS completed structured, self-administered, baseline surveys that gathered demographic, lifestyle, medical, reproductive, and family history information. Participants reported their birthweight as one of the following: <6 pounds (lbs.), 6 lbs. to 7 lbs. 15 ounces (oz.), 8 lbs. to 9 lbs. 15 oz., and ≥ 10 lbs. Validation of the collection of birthweight by category has been previously validated (Spearman *r* = 0.67–0.75) [19, 20]. Women were also asked to report if they were born preterm through the following question: “When you were born, were you: full term, 4 or more weeks premature, don’t know?” (Supplementary Table S1). Women also reported if they were part of a multiple pregnancy (e.g., a twin or triplet). Physical assessments to collect accurate anthropometric and other clinical measures were also performed at baseline by trained clinical staff.

### Outcome definitions and measurement

Questionnaires self-administered at baseline were used to obtain data on relevant health outcomes (Supplementary Table S1). At baseline, women were asked to report their age at menarche, as well as the ages they experienced their first regular period, last regular period, last vaginal bleeding, and menopause (further described below). Women were also asked whether they had ever had an oophorectomy (yes/no), or hysterectomy (yes/no); if yes, they were then asked at what age (< 30 to ≥ 60, in categories with 5-year increments). Additionally, women were asked if they ever had regular periods, premenstrual hot flashes, or postmenopausal hot flashes (yes/no). The reproductive window was calculated by subtracting the reported age of menarche from the age at natural menopause.

To be comparable to prior published literature, we restricted our menopause analyses (age at menopause, age at last vaginal bleeding, and age at last regular period) to women who experienced natural menopause. To define the age at natural menopause, we started with the primary age at menopause variable, as defined by WHI’s algorithm [21], which briefly defines a woman’s age at menopause as the age she first experienced any of the following: last time they had any menstrual bleeding, had a bilateral oophorectomy, or began using hormone replacement therapy. However, we further excluded participants who reported: (1) a history of bilateral oophorectomy at an age before their reported age at menopause; (2) a history of having an oophorectomy at an age before their reported age at menopause but did not know if it was unilateral or bilateral; or (3) had an unknown oophorectomy status. A second, more conservative age at natural menopause variable was used in sensitivity analyses, and further removed participants who reported having a of hysterectomy prior to their age at menopause or an unknown hysterectomy status.

**Table 1:** Baseline characteristics of 78,028 WHI study participants by birth weight category

		Birth weight category				
		< 6 lbs. *N* = 7,096	6 lbs.–7 lbs. 15 oz. *N* = 52,661	8 lbs.–9 lbs. 15 oz. *N* = 15,591	≥ 10 lbs. *N* = 2,680	*P* ^a^
Age at baseline (years)		63.1 (7.5)	63.3 (7.3)	63.4 (7.3)	65.0 (7.0)	< 0.0001
Race						
	Asian/Pacific Islander	348 (5.0)	1,349 (2.6)	171 (1.1)	21 (0.8)	< 0.0001
	Black	710 (10.3)	3,930 (7.6)	866 (5.7)	161 (6.1)	
	White	5,725 (83.0)	45,682 (88.5)	14,081 (91.9)	2,409 (91.6)	
	Other/Unknown	115 (1.7)	674 (1.3)	199 (1.3)	39 (1.5)	
Ethnicity						
	Hispanic/Latinx	363 (5.2)	2,096 (4.0)	509 (3.3)	84 (3.2)	< 0.0001
	Not Hispanic/Latinx	6,620 (94.8)	50,101 (96.0)	14,976 (96.7)	2,574 (96.8)	
Region						
	Northeast	1,518 (21.4)	11,977 (22.7)	3,424 (22.0)	572 (21.3)	<0.0001
	South	1,928 (27.2)	13,452 (25.5)	3,979 (25.5)	692 (25.8)	
	Midwest	1,516 (21.4)	3,979 (25.5)	3,638 (23.3)	642 (24.0)	
	West	2,134 (30.1)	692 (25.8)	4,550 (29.2)	774 (28.9)	
Education						
	≤ High school diploma/GED	1,642 (23.4)	10,423 (19.9)	2,981 (19.3)	664 (25.1)	< 0.0001
	School after high school	2,687 (38.3)	18,987 (36.3)	5,748 (37.2)	1,071 (40.4)	
	College degree or higher	2,695 (38.4)	22,875 (43.8)	6,735 (43.6)	916 (34.6)	
Normalized Socioeconomic Status (NSES)		75.3 (9.0)	76.2 (8.4)	76.4 (8.0)	75.4 (8.3)	< 0.0001
BMI at baseline (kg/m^2^)		27.3 (6.0)	27.0 (5.7)	27.7 (6.1)	28.6 (6.6)	< 0.0001
Smoking status						
	Never	3,705 (53.0)	26,268 (50.5)	7,500 (48.7)	1,304 (49.4)	< 0.0001
	Past	2,793 (40.0)	22,517 (43.3)	6,894 (44.8)	1,163 (44.1)	
	Current	493 (7.1)	3,195 (6.1)	1,003 (6.5)	173 (6.6)	
Alcohol Use						
	Non-drinker	933 (13.2)	5,459 (10.4)	1,513 (9.8)	321 (12.1)	< 0.0001
	Past drinker	1,484 (21.1)	9,418 (18.0)	2,827 (18.3)	551 (20.7)	
	Current drinker	4,628 (65.7)	37,466 (71.6)	11,153 (72.0)	1,787 (67.2)	
Participant breastfed						
	Yes	3,832 (67.4)	30,743 (72.7)	10,177 (77.2)	1,853 (82.3)	< 0.0001
	No	1,856 (32.6)	11,551 (27.3)	3,009 (22.8)	400 (17.8)	
Age at menarche (years)						
	≤ 11	1,701 (24.1)	11,538 (22.0)	3,517 (22.6)	637 (23.9)	< 0.0001
	12	1,816 (25.7)	13,813 (26.3)	4,029 (25.9)	670 (25.1)	
	13	1,916 (27.1)	15,466 (29.5)	4,526 (29.1)	713 (26.7)	
	14	1,635 (23.1)	11,628 (22.2)	3,469 (22.3)	646 (24.2)	
Age at first regular period (years)						
	≤ 11	908 (15.4)	6,034 (13.7)	1,920 (14.7)	351 (16.1)	< 0.0001
	12	1,242 (21.1)	9,125 (20.7)	2,719 (20.8)	444 (20.3)	
	13	1,405 (23.8)	11,598 (26.3)	3,395 (25.9)	555 (25.4)	
	>= 14	2,343 (39.7)	17,351 (39.3)	5,063 (38.7)	836 (38.2)	
Reproductive window (years)		36.1 (6.4)	36.6 (5.9)	36.6 (5.9)	36.1 (6.4)	< 0.0001
Age at last regular period (years)		46.9 (6.9)	47.5 (6.6)	47.5 (6.6)	47.4 (6.8)	< 0.0001
Age at last vaginal bleeding (years)		47.8 (7.5)	48.5 (7.3)	48.5 (7.3)	48.3 (7.4)	< 0.0001
Age at natural menopause (years)		48.6 (6.2)	49.1 (5.8)	49.2 (5.8)	48.8 (6.2)	< 0.0001
Were periods regular						
	Yes	5,663 (80.4)	43,395 (82.9)	12,736 (82.3)	2,139 (80.5)	< 0.0001
	No	1,384 (19.6)	8,978 (17.1)	2,743 (17.7)	519 (19.5)	
Ever had a hysterectomy						
	Yes	3,117 (44.0)	21,545 (41.0)	6,521 (41.9)	1,240 (46.3)	< 0.0001
	No	3,974 (56.0)	31,068 (56.0)	9,054 (58.1)	1,438 (53.7)	
Ever had an oophorectomy						
	Yes	2,200 (31.5)	14,921 (28.7)	4,528 (29.4)	848 (32.2)	< 0.0001
	No	4,791 (68.5)	37,057 (71.3)	10,889 (70.6)	1,788 (67.8)	
Ever had premenopausal hot flashes						
	Yes	5,857 (82.6)	43,902 (83.4)	13,064 (83.9)	2,242 (83.8)	0.10
	No	1,236 (17.4)	8,717 (16.6)	2,511 (16.1)	435 (16.3)	
Ever had postmenopausal hot flashes						
	Yes	2,355 (49.4)	17,531 (49.4)	5,171 (49.1)	882 (48.0)	0.61
	No	2,417 (50.7)	17,928 (50.6)	5,366 (50.9)	957 (52.0)	

Numbers are N (%) for categorical variables and mean (STD) for continuous variables. ^a^P values are from ANOVA and chi-square statistics and compare groups across birth weight categories

### Exclusion criteria

**Fig. 1:**
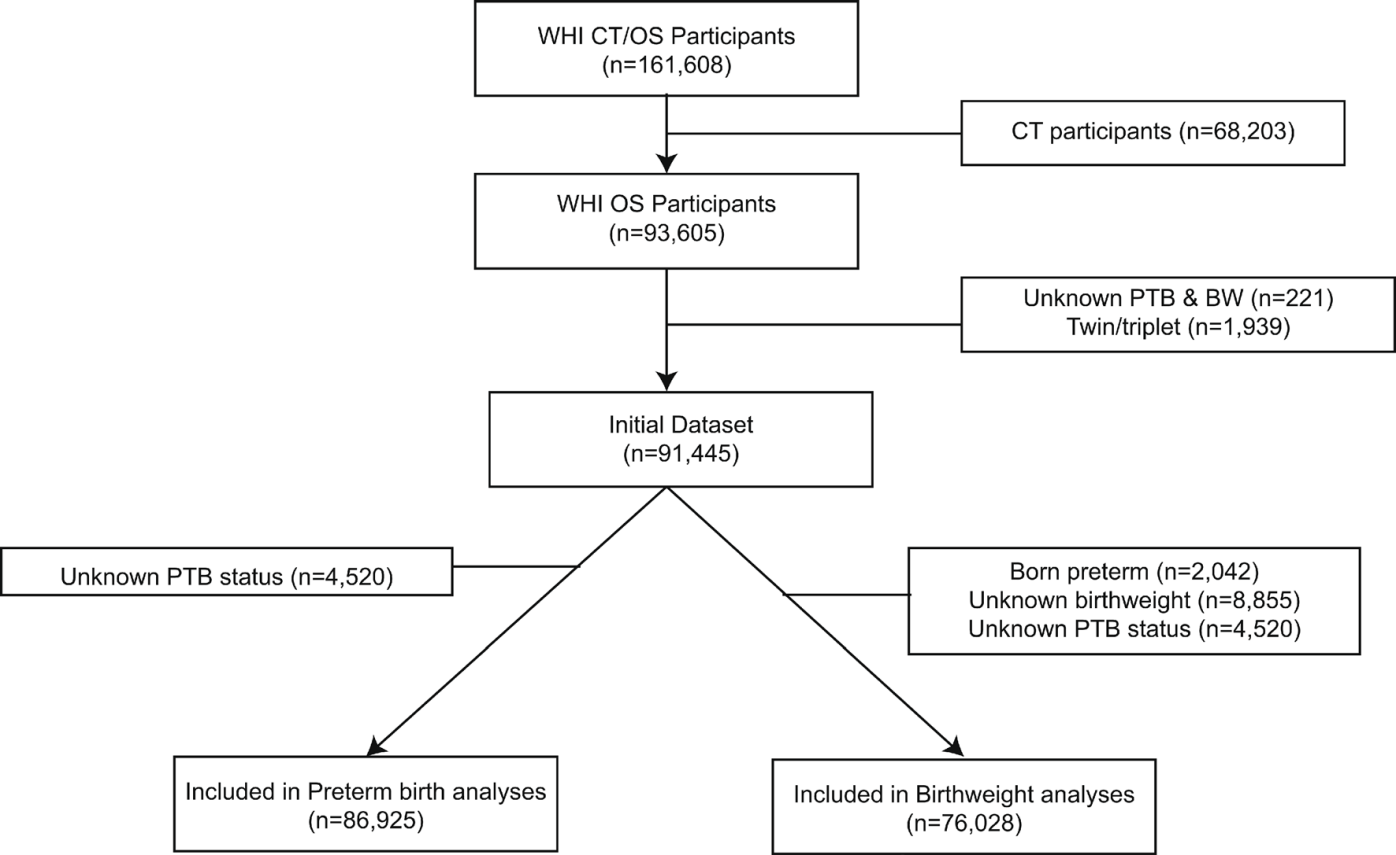
Flow chart for inclusion

Participants were excluded from all analyses if they were reported being a twin or triplet (*n* = 1,939) or if they were missing both birthweight information and premature birth status information (*n* = 2,924). Participants born ≥ 4 weeks premature were excluded from birthweight analyses (*n* = 2,042). Women who did not experience natural menopause were excluded from menopause-related analyses in both birthweight (*n* = 16,692) and preterm birth (*n* = 17,488) analyses. The final maximal sample size for the preterm birth analyses was *n* = 86,925 and the final maximal sample size for birthweight analyses was *n* = 76,028 (Fig. 1).

### Statistical analyses

Birthweight category (< 6 lbs., 6 lbs. to 7 lbs. 15 oz., 8 lbs. to 9 lbs. 15 oz., alownd ≥ 10 lbs.) and preterm birth status (≥ 4 weeks premature or full term) were used to examine baseline characteristics of study participants. For both analyses, categorical variables were examined using chi-square tests. Continuous variables were examined for using t-tests for preterm birth analyses and ANOVAs for birthweight analyses. Linear regression models were used to generate estimates between a participant’s birthweight or preterm birth status with their age at events related to menarche and menopause. Logistic regression models were used to estimate odds ratios (OR) and 95% confidence intervals (95% CI) between a woman’s preterm birth status or birthweight and regular periods, pre- and postmenopausal hot flashes, oophorectomy, and hysterectomy. The referent category for birthweight analyses was 6 lbs. to 7 lbs. 15 oz., as infants born full term and within this weight range are considered to be of normal weight. Full term was used as the referent category in preterm birth analyses. Covariates for model inclusion included age (continuous), race (categorical; Asian/Pacific Islander, Black, White, Other/Unknown), ethnicity (categorical; Hispanic/Latinx or non-Hispanic/Latinx), region (categorical), normalized neighborhood socio-economic status (NSES, continuous) [22], education (categorical; ≤ high school diploma/GED, school after high school, college degree or higher), baseline body mass index (BMI, continuous), smoking status (categorical; never, past, current), and alcohol use (categorical; non-drinker, past drinker, current drinker). Whether or not the participant was breast fed was also explored as a potential covariate; however, all effect estimates were unchanged in the analyses when added to the models and it was removed. We also stratified our results by race or ethnicity and provide the results in the supplementary materials; however, analyses of individuals who identify as non-White and Hispanic/Latinx are too underpowered to draw any conclusions. Further, we performed sensitivity analyses stratified by age group at enrollment for consideration of results among those who more recently experienced the hormonal transitional period.

**Table 2:** Results from linear regression analyses for the associations between birthweight and age at events related to menarche and menopause

			Birth weight category β (SE) *P*				
			< 6 lbs. *N* = 7,096	6 lbs.–7 lbs. 15 oz. *N* = 52,661	8 lbs.–9 lbs. 15 oz. *N* = 15,591	≥ 10 lbs. *N* = 2,680	Global *P*
*Menarche-related*							
	Age at menarche						
		Unadjusted (*n*=77,720)	-0.041 (0.02) 0.03	Ref	-0.023 (0.01) 0.09	0.023 (0.03) 0.42	0.049
		Adj. for demographics (*n*=74,840)	-0.044 (0.02) 0.02	Ref	0.005 (0.01) 0.72	0.046 (0.03) 0.12	< 0.0001
		Adj. for demographic and lifestyle factors (*n*=65,447)	-0.047 (0.02) 0.02	Ref	-0.003 (0.01) 0.82	0.032 (0.03) 0.31	< 0.0001
	Age at first regular period						
		Unadjusted (*n*=65,289)	-0.026 (0.03) 0.31	Ref	-0.043 (0.02) 0.02	-0.050 (0.04) 0.21	0.07
		Adj. for demographics (*n*=62,910)	-0.016 (0.03) 0.54	Ref	-0.017 (0.02) 0.36	-0.011 (0.04) 0.79	< 0.0001
		Adj. for demographic and lifestyle factors (*n*=55,274)	-0.011 (0.03) 0.68	Ref	-0.016 (0.02) 0.42	-0.026 (0.04) 0.54	< 0.0001
*Reproductive window*							
	Reproductive window (years)						
		Unadjusted (*n*=54,348)	-0.455 (0.09) < 0.0001	Ref	0.058 (0.07) 0.37	-0.409 (0.14) 0.004	< 0.0001
		Adj. for demographics (*n*=52,445)	-0.403 (0.09) < 0.0001	Ref	0.034 (0.07) 0.61	-0.466 (0.14) 0.001	< 0.0001
		Adj. for demographic and lifestyle factors (*n*=46,036)	-0.287 (0.10) 0.004	Ref	0.029 (0.07) 0.67	-0.283 (0.15) 0.06	< 0.0001
*Menopause-related*							
	Age at last regular period						
		Unadjusted (*n*=70,263)	-0.585 (0.09) < 0.0001	Ref	0.042 (0.06) 0.51	-0.135 (0.14) 0.33	< 0.0001
		Adj. for demographics (*n*=67,805)	-0.528 (0.09) < 0.0001	Ref	0.036 (0.06) 0.57	-0.217 (0.14) 0.12	< 0.0001
		Adj. for demographic and lifestyle factors (*n*=59,460)	-0.445 (0.09) < 0.0001	Ref	0.063 (0.07) 0.35	-0.083 (0.15) 0.57	< 0.0001
	Age at last vaginal bleeding						
		Unadjusted (*n*=71,538)	-0.717 (0.10) < 0.0001	Ref	0.034 (0.07) 0.62	-0.214 (0.15) 0.16	< 0.0001
		Adj. for demographics (*n*=69,009)	-0.631 (0.10) < 0.0001	Ref	0.027 (0.07) 0.70	-0.311 (0.15) 0.04	< 0.0001
		Adj. for demographic and lifestyle factors (*n*=60,492)	-0.524 (0.10) < 0.0001	Ref	0.048 (0.07) 0.52	-0.151 (0.16) 0.35	< 0.0001
	Age at natural menopause						
		Unadjusted (*n*=62,015)	-0.494 (0.08) < 0.0001	Ref	0.060 (0.06) 0.32	-0.308 (0.13) 0.02	< 0.0001
		Adj. for demographics (*n*=59,822)	-0.450 (0.09) < 0.0001	Ref	0.060 (0.06) 0.32	-0.342 (0.13) 0.01	< 0.0001
		Adj. for demographic and lifestyle factors (*n*=52,416)	-0.350 (0.09) < 0.0001	Ref	0.059 (0.06) 0.35	-0.159 (0.14) 0.25	< 0.0001
	Age at natural menopause (conservative)						
		Unadjusted (*n*=54,413)	-0.478 (0.09) < 0.0001	Ref	0.041 (0.06) 0.52	-0.361 (0.14) 0.01	< 0.0001
		Adj. for demographics (*n*=52,506)	-0.432 (0.09) < 0.0001	Ref	0.043 (0.06) 0.50	-0.391 (0.14) 0.01	< 0.0001
		Adj. for demographic and lifestyle factors (*n*=46,084)	-0.317 (0.10) 0.001	Ref	0.035 (0.07) 0.61	-0.219 (0.15) 0.14	< 0.0001

Results presented as beta (standard error) and p-value. Global P-value is testing for a linear tread. Demographic factors include age, race, ethnicity, region, and BMI. Lifestyle factors include smoking status, education, normalized socioeconomic status (NSES), and alcohol use. For the age at natural menopause analyses, participants were removed if they reported: having a bilateral oophorectomy prior to menopause; having an oophorectomy prior to menopause but did not know if it was unilateral or bilateral; or if they had an unknown oophorectomy status. For the conservative age at natural menopause, participants were also removed if they reported having a hysterectomy prior to menopause or had an unknown hysterectomy status

In the field of life-course epidemiology, there is some controversy as to whether or not adult lifestyle factors (e.g., BMI) should be included as model covariates [23]. As such, we present our results unadjusted, partially adjusted for demographic factors, and fully adjusted for demographic and lifestyle factors that may have occurred temporally after our exposure. Furthermore, we did not include birthweight as a covariate in preterm birth analyses, as birthweight and gestational age are strongly correlated and would have potentially adjusted away part or all of the association between preterm birth and the outcomes of interest. Instead, results are presented stratified by birthweight category to allow for a more direct comparisons of disease risk among women born in similar birthweights; this also allows for examination of preterm birth analyses among those individuals most likely born preterm. All statistical tests were two-sided, with P-values < 0.05 being considered statistically significant. All analyses were performed using R (Version 4.1.2).

**Table 3:** Results from logistic regression analyses for the associations between birthweight and events related to menarche and menopause

			Birth weight category OR (95% CI) *P*				
			< 6 lbs. *N* = 7,093	6 lbs.–7 lbs. 15 oz. *N* = 52,619	8 lbs.–9 lbs. 15 oz. *N* = 15,575	≥ 10 lbs. *N* = 2,677	Global *P*
*Menarche-related*							
	Were periods regular						
		Unadjusted (*n*=77,584)	0.85 (0.80-0.90) < 0.0001	1.00 (Ref)	0.96 (0.92-1.01) 0.11	0.85 (0.77-0.94) 0.002	< 0.001
		Adj. for demographics (*n*=74,707)	0.84 (0.79-0.90) < 0.0001	1.00 (Ref)	0.97 (0.92-1.02) 0.18	0.85 (0.77-0.94) 0.002	< 0.001
		Adj. for demographic and lifestyle factors (*n*=65,332)	0.84 (0.78-0.90) < 0.0001	1.00 (Ref)	0.97 (0.92-1.02) 0.24	0.86 (0.77-0.96) 0.007	< 0.001
*Menopause-related*							
	Ever had premenopausal hot flashes						
		Unadjusted (*n*=77,964)	0.94 (0.88-1.01) 0.07	1.00 (Ref)	1.03 (0.98-1.08) 0.19	1.02 (0.92-1.14) 0.67	0.10
		Adj. for demographics (*n*=75,069)	0.95 (0.89-1.02) 0.13	1.00 (Ref)	1.03 (0.98-1.08) 0.27	0.99 (0.89-1.10) 0.79	< 0.0001
		Adj. for demographic and lifestyle factors (*n*=65,601)	0.96 (0.89-1.03) 0.27	1.00 (Ref)	1.02 (0.97-1.08) 0.47	1.03 (0.92-1.16) 0.61	< 0.0001
	Ever had postmenopausal hot flashes						
		Unadjusted (*n*=52,607)	1.00 (0.94-1.06) 0.91	1.00 (Ref)	0.99 (0.94-1.03) 0.51	0.94 (0.86-1.04) 0.22	0.61
		Adj. for demographics (*n*=50,673)	1.00 (0.94-1.07) 0.99	1.00 (Ref)	0.98 (0.94-1.02) 0.36	1.00 (0.90-1.10) 0.92	0.83
		Adj. for demographic and lifestyle factors (*n*=44,300)	1.00 (0.94-1.07) 0.97	1.00 (Ref)	0.99 (0.94-1.04) 0.57	0.98 (0.88-1.08) 0.63	0.92
	Ever had an oophorectomy						
		Unadjusted (*n*=77,022)	1.14 (1.08-1.20) < 0.0001	1.00 (Ref)	1.03 (0.99-1.07) 0.11	1.18 (1.08-1.28) 0.0001	< 0.0001
		Adj. for demographics (*n*=74,190)	1.15 (1.08-1.21) < 0.0001	1.00 (Ref)	1.02 (0.98-1.07) 0.25	1.13 (1.03-1.23) 0.007	< 0.0001
		Adj. for demographic and lifestyle factors (*n*=64,888)	1.14 (1.07-1.21) < 0.0001	1.00 (Ref)	1.03 (0.98-1.07) 0.25	1.13 (1.03-1.24) 0.008	< 0.0001
	Ever had a hysterectomy						
		Unadjusted (*n*=77,957)	1.13 (1.08-1.19) < 0.0001	1.00 (Ref)	1.04 (1.00-1.08) 0.04	1.24 (1.15-1.34) < 0.0001	< 0.0001
		Adj. for demographics (*n*=75,067)	1.13 (1.08-1.19) < 0.0001	1.00 (Ref)	1.03 (0.99-1.07) 0.18	1.17 (1.08-1.26) 0.0002	< 0.0001
		Adj. for demographic and lifestyle factors (*n*=65,558)	1.11 (1.05-1.18) 0.0002	1.00 (Ref)	1.02 (0.98-1.06) 0.28	1.15 (1.05-1.25) 0.002	< 0.0001

Results presented as odds ratio (95% confidence interval) and p-value. Demographic factors include age, race, ethnicity, region, and BMI. Lifestyle factors include smoking status, education, normalized socioeconomic status (NSES), and alcohol use

**Table 4:** Baseline characteristics of 86,925 WHI study participants by preterm birth status

		Preterm birth *N* = 2,042	Full term *N* = 84,883	*P* ^a^
Age at baseline (years)		62.0 (7.3)	63.5 (7.4)	< 0.0001
Race				
	Asian/Pacific Islander	44 (2.2)	2,327 (2.8)	0.43
	Black	162 (8.1)	6,621 (8.0)	
	White	1,770 (88.4)	72,954 (87.9)	
	Other/Unknown	26 (1.3)	1,135 (1.4)	
Ethnicity				
	Hispanic/Latinx	83 (4.1)	3,588 (4.3)	0.71
	Not Hispanic/Latinx	1,941 (95.9)	80,472 (95.7)	
Region				
	Northeast	425 (20.8)	19,296 (22.7)	0.23
	South	548 (26.8)	22,004 (25.9)	
	Midwest	462 (22.6)	18,788 (22.1)	
	West	607 (29.7)	24,795 (29.2)	
Education				
	≤ High school diploma/GED	363 (17.9)	17,715 (21.0)	0.002
	School after high school	744 (36.7)	30,679 (36.4)	
	College degree or higher	918 (45.3)	35,820 (42.5)	
Normalized Socioeconomic Status (NSES)		75.8 (8.2)	76.0 (8.6)	0.26
BMI at baseline (kg/m^2^)		27.7 (6.1)	27.2 (5.9)	0.0001
Smoking status				
	Never	1,039 (51.6)	42,422 (50.6)	0.09
	Past	828 (41.2)	36,110 (43.1)	
	Current	145 (7.2)	5,246 (6.3)	
Alcohol Use				
	Non-drinker	218 (10.7)	9,316 (11.1)	0.31
	Past drinker	406 (20.0)	15,709 (18.6)	
	Current drinker	1,410 (69.3)	59,310 (70.3)	
Participant breast fed				
	Yes	882 (53.6)	50,863 (74.4)	< 0.0001
	No	765 (46.5)	17,513 (25.6)	
Birthweight				
	< 6 lbs.	1,687 (84.6)	6,359 (8.4)	< 0.001
	6 lbs. – 7 lbs. 15 oz.	277 (13.9)	51,728 (67.9)	
	8 lbs. – 9 lbs. 15 oz.	29 (1.5)	15,414 (20.2)	
	≥ 10 lbs.	< 20 (<1.0)	2,638 (3.5)	
Age at menarche (years)				
	≤ 11	502 (24.7)	18,646 (22.1)	0.03
	12	527 (25.9)	22,112 (26.2)	
	13	551 (27.1)	24,571 (29.1)	
	14	452 (22.2)	19,214 (22.7)	
Age at first regular period (years)				
	≤ 11	253 (15.3)	9,903 (14.0)	0.46
	12	343 (20.8)	14,742 (20.8)	
	13	424 (25.7)	18,418 (26.0)	
	14	633 (38.3)	27,900 (39.3)	
Age at last regular period (years)		46.8 (6.8)	47.5 (6.6)	< 0.0001
Age at last vaginal bleeding (years)		47.7 (7.6)	48.4 (7.3)	< 0.0001
Were periods regular				
	Yes	1,599 (78.9)	69,651 (82.5)	< 0.0001
	No	427 (21.1)	14,730 (17.5)	
Ever had a hysterectomy				
	Yes	889 (43.6)	35,297 (41.6)	0.08
	No	1,151 (56.4)	49,512 (58.4)	
Ever had an oophorectomy				
	Yes	644 (31.9)	24,483 (29.2)	0.009
	No	1,373 (68.1)	59,269 (70.8)	
Age at natural menopause (years)		48.4 (6.3)	49.1 (5.9)	< 0.0001
Ever had premenopausal hot flashes				
	Yes	1,682 (82.4)	70,802 (83.5)	0.18
	No	360 (17.6)	14,013 (16.5)	
Ever had postmenopausal hot flashes				
	Yes	687 (51.4)	28,807 (49.0)	0.09
	No	651 (48.7)	29,296 (51.1)	
Reproductive window (years)		35.9 (6.3)	36.5 (6.0)	< 0.0001

Numbers are N (%) for categorical variables and mean (STD) for continuous variables. ^a^P values are from ANOVA and chi-square statistics and compare groups across birth weight categories. Policy from the Women’s Health Initiative will not allow researchers to report the number of participants in cells with fewer than 20 individuals. As such, cells that contain fewer than 20 participants read “<20”

## Results

### Birthweight

Baseline characteristics of participants stratified by birthweight category are shown in Table 1. Within the final study sample, 9.1%, 67.4%, 20.0%, and 3.4% of participants were reportedly born within the < 6 lbs., 6 lbs.-7 lbs. 15 oz., 8 lbs. – 9 lbs. 15 oz, or ≥ 10 lbs. categories, respectively. The average ages at menarche, natural menopause, and natural menopause (conservative) for the entire study population was 12.2, 49.0, and 49.1 years. Participants who reported weighing ≥ 10 lbs. at birth were more likely to be older and have a higher BMI at baseline. They were also more likely to report being breast fed as an infant, have a hysterectomy prior to enrollment, and experience postmenopausal hot flashes. Individuals who reported weighing < 6 lbs. at birth were more likely to be younger at baseline, identify as Black or Hispanic/Latinx, and identify as a never-smoker and/or non-drinker at enrollment.

**Table 5:** Results from linear regression analyses for the associations between preterm birth status and age at events related to menarche and menopause

			Preterm birth β (SE) *N* = 2,042	Full term β (SE) *N* = 84,883	*P*
*Menarche-related*					
	Age at first regular period				
		Unadjusted (*n*=72,616)	-0.037 (0.02)	Ref	0.17
		Adj. for demographics (*n*=79,796)	-0.024 (0.03)	Ref	0.36
		Adj. for demographic and lifestyle factors (*n*=61,348)	-0.022 (0.03)	Ref	0.43
	Age at menarche				
		Unadjusted (*n*=86,575)	-0.055 (0.02)	Ref	0.02
		Adj. for demographics (*n*=83,156)	-0.026 (0.02)	Ref	0.28
		Adj. for demographic and lifestyle factors (*n*=72,719)	-0.031 (0.03)	Ref	0.23
*Reproductive window*					
	Reproductive window (years)				
		Unadjusted (*n*=60,412)	-0.629 (0.16)	Ref	< 0.0001
		Adj. for demographics (*n*=58,165)	-0.532 (0.17)	Ref	0.001
		Adj. for demographic and lifestyle factors (*n*=51,037)	-0.418 (0.17)	Ref	0.006
*Menopause-related*					
	Age at last regular period				
		Unadjusted (*n*=78,268)	-0.624 (0.16)	Ref	< 0.0001
		Adj. for demographics (*n*=75,352)	-0.446 (0.16)	Ref	0.005
		Adj. for demographic and lifestyle factors (*n*=66,082)	-0.397 (0.17)	Ref	0.02
	Age at last vaginal bleeding				
		Unadjusted (*n*=79,664)	-0.746 (0.17)	Ref	< 0.0001
		Adj. for demographics (*n*=76,671)	-0.533 (0.17)	Ref	0.002
		Adj. for demographic and lifestyle factors (*n*=67,194)	-0.435 (0.18)	Ref	0.02
	Age at natural menopause				
		Unadjusted (*n*=68,889)	-0.682 (0.15)	Ref	< 0.0001
		Adj. for demographics (*n*=66,291)	-0.531 (0.15)	Ref	0.0004
		Adj. for demographic and lifestyle factors (*n*=58,084)	-0.506 (0.16)	Ref	0.001
	Age at natural menopause (conservative)				
		Unadjusted (*n*=60,487)	-0.681 (0.16)	Ref	< 0.0001
		Adj. for demographics (*n*=58,234)	-0.543 (0.16)	Ref	0.0008
		Adj. for demographic and lifestyle factors (*n*=51,092)	-0.493 (0.17)	Ref	0.004

Results presented as beta (standard error). Demographic factors include age, race, ethnicity, region, and BMI. Lifestyle factors include smoking status, education, normalized socioeconomic status (NSES), and alcohol use. For the age at natural menopause analyses, participants were removed if they reported: having a bilateral oophorectomy prior to menopause; having an oophorectomy prior to menopause but did not know if it was unilateral or bilateral; or if they had an unknown oophorectomy status. For the conservative age at natural menopause, participants were also removed if they reported having a hysterectomy prior to menopause or had an unknown hysterectomy status

Table 2 shows the crude and adjusted estimates for the associations between birthweight and age at menarche and menopause. After adjustment for demographic and lifestyle, participants born weighing < 6 lbs. reached natural menopause at an earlier age compared to those born weighing 6 lbs. – 7 lbs. 15 oz in models using the standard (adjβ = -0.350, *p* < 0.0001; 4.3 months) and conservative (adjβ = -0.317, *p* < 0.0001; 3.9 months) definitions of natural menopause. Being born weighing < 6 lbs. was also associated with a shorter reproductive window compared to those born weighing 6 lbs. − 7 lbs. 15 oz (adjβ = -0.287, *p* = 0.004; 3.5 months). We also observed individuals born weighing < 6 lbs. having a lower age at menarche (adjβ = -0.047, *p* = 0.02; 0.56 months). Individuals born weighing ≥ 10 lbs. were also more likely to experience an earlier age at menopause with both definitions and have a shorter reproductive window, but the significant associations were attenuated in the fully adjusted models. Results stratified by age, race, and ethnicity are shown in Supplementary Tables 2 and 3.

**Table 6:** Results from logistic regression analyses for the associations between preterm birth status and events related to menarche and menopause

			Preterm birth OR (95% CI) *N* = 2,042	Full Term birth OR (95% CI) *N* = 84,815	*P*
*Menarche-related*					
	Were periods regular				
		Unadjusted (*n*=86,407)	0.79 (0.71-0.88)	1.00 (Ref)	< 0.0001
		Adj. for demographics (*n*=82,992)	0.82 (0.73-0.91)	1.00 (Ref)	0.0003
		Adj. for demographic and lifestyle factors (*n*=72,581)	0.81 (0.72-0.92)	1.00 (Ref)	0.0007
*Menopause-related*					
	Ever had premenopausal hot flashes				
		Unadjusted (*n*=86,857)	0.93 (0.82-1.04)	1.00 (Ref)	0.18
		Adj. for demographics (*n*=83,418)	0.94 (0.83-1.05)	1.00 (Ref)	0.27
		Adj. for demographic and lifestyle factors (*n*=72,898)	0.96 (0.85-1.09)	1.00 (Ref)	0.52
	Ever had postmenopausal hot flashes				
		Unadjusted (*n*=58,721)	1.10 (0.99-1.23)	1.00 (Ref)	0.08
		Adj. for demographics (*n*=56,411)	1.04 (0.93-1.17)	1.00 (Ref)	0.45
		Adj. for demographic and lifestyle factors (*n*=49,326)	1.05 (0.93-1.18)	1.00 (Ref)	0.47
	Ever had an oophorectomy				
		Unadjusted (*n*=85,769)	1.14 (1.03-1.25)	1.00 (Ref)	0.009
		Adj. for demographics (*n*=82,409)	1.15 (1.05-1.27)	1.00 (Ref)	0.004
		Adj. for demographic and lifestyle factors (*n*=72,078)	1.13 (1.01-1.25)	1.00 (Ref)	0.03
	Ever had a hysterectomy				
		Unadjusted (*n*=86,849)	1.08 (0.99-1.18)	1.00 (Ref)	0.07
		Adj. for demographics (*n*=83,414)	1.08 (0.99-1.18)	1.00 (Ref)	0.10
		Adj. for demographic and lifestyle factors (*n*=72,850)	1.07 (0.97-1.18)	1.00 (Ref)	0.19

Results presented as odds ratio (95% confidence interval). Demographic factors include age, race, ethnicity, region, and BMI. Lifestyle factors include smoking status, education, normalized socioeconomic status (NSES), and alcohol use

Table 3 shows the unadjusted and demographic- and lifestyle-adjusted odds ratios for events related to menarche and menopause by category of birthweight. Compared to those born weighing 6 lbs-7 lbs. 15 oz, participants born weighing < 6 lbs. and those born weighing ≥ 10 lbs. were significantly less likely to report having regular menstrual periods (< 6 lbs: adjOR = 0.84, 95% CI 0.78–0.90; ≥10 lbs.: adjOR = 0.86, 95% CI 0.77–0.96). Furthermore, participants born weighing < 6 lbs. and ≥ 10 lbs. had significantly higher odds of reporting a prior oophorectomy (< 6 lbs.: adjOR = 1.14, 95% CI 1.07–1.21; ≥10 lbs.: adjOR = 1.13, 95% CI 1.03–1.24) and hysterectomy (< 6 lbs.: adjOR = 1.11, 95% CI 1.05–1.18; ≥10 lbs.: adjOR = 1.15, 95% CI 1.05–1.25) at study enrollment, compared to those born weighing 6 lbs-7 lbs. 15 oz. Results stratified by age, race, and ethnicity are shown in Supplementary Tables 4 and 5.

### Preterm Birth

Baseline characteristics of participants stratified by preterm birth status are shown in Table 4. Within the final study sample, 2.3% of participants were reportedly born 4 or more weeks preterm. At enrollment, participants who reported being born preterm were more likely to be younger and have a higher BMI. They were also more likely to identify as White, identify as Not Hispanic/Latinx, have a college degree or higher, weigh less than 6 lbs. at birth, be formula fed as an infant, have had an oophorectomy, and report a younger age at menopause and last regular period.

Table 5 shows the crude and adjusted estimates for the associations between a participant’s preterm birth status and continuous variables related to menarche and menopause. Preterm birth was not associated with an earlier age at menarche (adjβ = -0.031, *p* = 0.23). When compared to participants born full term, individuals born preterm experienced a shorter reproductive window (adjβ = -0.418, *p* = 0.006; 5.1 months), earlier natural menopause (adjβ = -0.506, *p* = 0.001; 6.2 months), had their last regular period earlier (adjβ = -0.397, *p* = 0.02; 4.9 months), experienced their last vaginal bleeding earlier (adjβ = -0.435, *p* = 0.02; 5.3 months), and had a shorter reproductive window (adjβ = -0.418, *p* = 0.006; 5.1 months). However, when restricted to participants born weighing < 6 lbs. (those individuals representing the most likely preterm individuals), there were no associations between preterm birth status and any of continuous variables related to menarche and menopause (Supplemental Table 6), suggesting that the birthweight category is a likely mediator in the observed associations with preterm birth status. Results stratified by age, race, and ethnicity are shown in Supplementary Tables 7 and 8.

Table 6 shows the odds ratios for events related to menarche and menopause by participant preterm birth status. Individuals born preterm had lower odds of having regular periods (adjOR = 0.81, 95% CI 0.72–0.92) compared to those born full term. Additionally, individuals born preterm had higher odds of having an oophorectomy prior to study enrollment (adjOR = 1.13, 95% CI 1.01–1.25) compared to those born full term. No associations were observed between preterm birth status and the odds of having a hysterectomy prior to enrollment, premenopausal hot flashes, or postmenopausal hot flashes. Importantly, when limiting analyses to women born in the lowest birthweight category (< 6 lbs.), no associations were observed for any of the outcomes (Supplemental Table 9), suggesting that the birthweight category is a likely mediator in the observed associations with preterm birth status and regular periods and oophorectomy prior enrollment. Results stratified by age, race, and ethnicity are shown in Supplementary Tables 10 and 11.

## Discussion

Our study of postmenopausal women from the WHI identified several meaningful associations between an individual’s birthweight and preterm birth status with outcomes relating to menarche and menopause. Individuals born weighing < 6 lbs. were more likely to have an earlier age at natural menopause when compared to normal birthweight; have a shorter reproductive window; experience irregular periods; and report an oophorectomy and/or hysterectomy prior to enrollment. Further, our results suggest a non-linear relationship between birthweight and age at natural menopause, where those born within the highest and lowest birthweight categories are more likely to experience an earlier age at natural menopause. Women born prematurely were more likely to experience natural menopause at an earlier age and with an overall shorter reproductive window.

The observed association between lower birthweight and earlier age of menarche and menopause has previously been reported. In a systematic review, 10/13 studies (76.9%) observed a statistically significant association between being born at lower birthweights and earlier menarcheal age; all studies included adjustment for potential confounders [8]. Similar to our study, two of the four previous studies that also excluded individuals born preterm identified earlier ages of menarche among those born in the lowest birthweight quintile (p for trend = 0.03) [24] or < 3,000 g (β = -0.68, *p* = 0.02) [25]. A similar number of studies have examined the association between birthweight and age at menopause; the majority did not observe a significant relationship [26]. However, most of the studies were very small (151 < *N* < 1,583), which could explain the null findings [26]. One study that was the most similar in design to ours, conducted in the Nurses’ Health Study II cohort, included data from > 106,000 US-based participants. Results from this study demonstrated an increased risk of early natural menopause among women born weighing < 5 lbs. 8 oz. (HR 1.21, 95% CI 1.01–1.45 in fully adjusted model) [27], consistent with our results. Future research in other diverse, non-US based populations is needed to confirm the observed relationships.

The prior literature examining the association between being born preterm and age of menarche are limited and inconsistent. There have been at least 13 studies that have previously considered the association between gestational age or being born preterm and age of menarche: 5 found preterm birth to be associated with earlier age of menarche and 8 found no association [28]. Among the four studies performed after the majority of the participants would have been expected to attain menarche (age > 15) [29–32], only one found individuals born preterm to achieve menarche earlier (beta = 0.07 years; *N* = 76,720; France) [30]. The remaining studies had a much smaller sample size (403 < *N* < 18,132). However, in our study of over 86,000 participants, we did not observe an association between preterm birth and age of menarche.

To our knowledge, only three studies have examined the association between preterm birth and age at menopause [16, 27, 33]. Specifically, two studies demonstrated preterm birth to be significantly associated with premature ovarian insufficiency [16, 33]. The third study from the Nurses’ Health Study II cohort found no association between preterm birth and age at natural menopause (OR 0.98, 95% CI 0.82–1.16) [27]. We are the first study to find an association between being born preterm and age at menopause. However, because our results were attenuated in models restricted to individuals who also reported weighing < 6 lbs. at birth, additional research in other populations would be needed to confirm the association in a dataset that does not rely on self-reported exposures.

Low birth weight, an established marker of poor infant health and/or nutrition [34], is thought to impact the age of menarche through alterations in the hypothalamic-pituitary-gonadal (HPG) axis. The HPG axis, which controls pubertal development [35], is established early in fetal development and remains active throughout pregnancy and through the first year of life [36, 37]. The HPG axis is then reactivated at pubertal onset [36, 37]. A poor intrauterine environment, caused by stress and/or inadequate nutrition, may cause fetal (FGR) or intrauterine (IUGR) growth restriction [8], which has been suggested to impact epigenetic programming [38, 39], including the development of the HPG axis and its reactivation at puberty [35]. Further, infants with suspected FGR experience catch-up growth, which has been shown to yield higher levels of BMI/adiposity, leptin and insulin-like growth factor 1 [40, 41] which are known to influence the gonadotropin (GnRH) system and have direct gonadal effects [42, 43]; thus impacting the onset of menarche.

Birthweight and preterm birth may also affect menopausal onset through mechanisms associated with fetal/intrauterine growth restriction. Determinants of the age at menopause include both the size of the ovarian follicle pool at birth and the rate of atresia [44]. In addition to its effect on the HPG axis, suboptimal *in utero* conditions may affect the formation of the initial cohort of ovarian follicles and fetal oocyte atresia [44, 45], leading to premature ovarian insufficiency [16] and, thereby, influencing the timing of menopause. Several animal studies have demonstrated that exposure to IUGR and low birth weight results in poorer ovarian volume and/or function [46–49], further supporting the biologic mechanism(s) by which low birth weight and/or preterm birth negatively impact age at menopause.

Our analyses were strengthened by our large sample size (> 76,000) and by the availability of a multitude of covariates to include in models such as race, socioeconomic status, BMI, and ethnicity. We were also able to exclude those born preterm from birthweight analyses and perform sensitivity analyses for models examining associations with preterm birth by stratifying the results by an individual’s birthweight.

It is somewhat difficult to apply our findings related to preterm birth to infants born today. Survival of infants born preterm is drastically improved in recent decades as a result of advancements in medical technology and pharmacologic interventions, including use of high frequency ventilators, surfactant therapy, corticosteroids, and prophylactic antibiotics, much of which was not widely used until the 1990s [50, 51]. During the 1910–1940’s when women in the WHI were born, survival of infants born very or extremely preterm was very low and only those with mild prematurity (or the “healthiest”) survived [50]. This overall survival is apparent even among our sample, as the prevalence of preterm birth increases with decreasing age at enrollment (45–54 years: 3.1%; ≥75 years: 1.7%). As a result, our results may not be directly relevant to those born preterm in recent years. However, it is becoming more common to see an overall reduction in the health and quality of life in very or extremely preterm infants who survive [52]. Therefore, if true, one would expect the associations observed in this study to be stronger among those individuals who did not survive to be included in our study [53] and to be relevant to those born in the current era.

Our study includes reliance on self-report for all exposures, outcomes, and potential covariates, which could have resulted in misclassification. While having birthweight and gestational age information as quantitative measures from birth records would have been preferred, self-reported birthweight and birthweight category has been previously validated among middle- and older-aged adults (Spearman *r* = 0.67-085) [19, 20, 54, 55], although there is demonstrated variability when stratifying by age (*r* = 0.80, 59–69 years; *r* = 0.85, 44–48 years) [55]; the validity of self-reported preterm birth status is unknown. However, we would expect any exposure misclassification within our study to be nondifferential and bias our results toward the null. Further, as our study participants were born in the 1910–1940’s, a time during which fetal ultrasounds were not available and gestational age was estimated solely based on last menstrual period and size of the infant at birth, self-reported preterm birth exposure is prone to an unknown degree of misclassification. While all outcome variables were self-reported, we would not expect any misclassification to be differential by birthweight or preterm birth status; as such, any bias introduced would be toward the null.

Additional limitations of our study warrant consideration. While the WHI is extensively phenotyped, information on additional covariates, including maternal smoking status, postnatal growth and childhood BMI, were not available, all of which are suspected to be related to age at menarche and menopause. Because gestational age and birthweight are highly correlated, our analyses may be confounded by birthweight as we were unable to adjust for this as a continuous variable; however, we did perform sensitivity analyses limiting preterm birth models to only those reportedly weighing < 6 lbs. to limit potential confounding and misclassification by self-report. It is also possible that excluding women who did not have a natural menopause from our analyses (i.e. those with a history or oophorectomy and/or hysterectomy) may have biased our results differentially in both the birthweight and preterm birth analyses. However, inclusion of these individuals would have resulted in a lower average age at menopause and biased our results away from the null. As a result, we chose to exclude these individuals from our menopause analyses. Further, while the WHI is a diverse cohort of individuals, analyses stratified by race or ethnicity were underpowered for all non-White and Hispanic/Latinx groups. Future studies in more diverse populations are warranted.

In conclusion, we found that being born low birthweight and/or preterm was associated with an earlier age of natural menopause. This research further supports the Barker hypothesis, expanding the list of later-in-life outcomes believed to be related to *in utero* exposures. Interventions aimed at preventing prematurity and low birthweight infants may reduce the number of individuals experiencing early menopause and, subsequently, may reduce the risk of associated comorbidities. Further, individuals known to be born at lower birthweights and premature may benefit from earlier counseling and/or intervention strategies aimed at reducing their risk for outcomes associated with earlier hormonal transition periods.

## Electronic supplementary material

Below is the link to the electronic supplementary material.

Supplementary Material 1

## Data Availability

Data from the Women’s Health Initiative is available upon request. Requests should be made to the WHI study coordinating center (p&p@whi.org).
