# Supplementary material for: Associations between birthweight and preterm birth and the ages at menarche and menopause
Source: BMC Womens Health. 2024 Oct 3;24:546. doi: 10.1186/s12905-024-03384-6 (PMC11448270; doi:10.1186/s12905-024-03384-6)
Supplement: Supplementary file 1 — Supplementary Material 1 [file 12905_2024_3384_MOESM1_ESM.docx]

**Supplementary File**

**Associations between birthweight and preterm birth and the ages at menarche and menopause**

Christian Daniele^1^, Rachel E. Wacks^1^, Leslie V. Farland^2^, JoAnn E. Manson^3,4^, Lihong Qi^5^, Aladdin H. Shadyab^6^, Sylvia Wassertheil-Smoller,^7^ Cassandra N. Spracklen^1^*

| **Supplementary Table 1: List of questions from the Women’s Health Initiative Observational Study used to measure exposures and outcomes for this study.** | | | | |
| --- | --- | --- | --- | --- |
| **Variable** | | **Form** | **Question** | **Answer Choices** |
| ***Exposures*** | |  |  |  |
|  | Birthweight | 42 | When you were born, about how much did you weigh? (Give your best guess.) | < 6 lbs.; 6 lbs. to 7 lbs. 15 oz; 8 lbs. to 9 lbs. 15 oz; ≥10 lbs.; Don’t Know |
|  | Preterm birth | 42 | When you were born, were you: full-term, premature, don’t know | Full term; 4 or more weeks premature; don’t know |
|  |  |  |  |  |
| ***Outcomes*** | |  |  |  |
|  | Age at menarche | 31 | How old were you when you had your first menstrual period (menses)? | ≤9; 10; 11; 12; 13; 14; 15; 16; ≥17 |
|  | Were periods regular | 31 | During most of your life, were your periods regular; that is, did they occur about once a month? (Do not include any time when you were pregnant or taking birth control pills.) | No; Yes; Sometimes regular, sometimes irregular |
|  | Age at first regular period | 31 | How old were you when your periods first became regular? (Your best guess) *Subquestion of “were periods regular” | ≤9; 10; 11; 12; 13; 14; 15; 16; ≥17 |
|  | Reproductive window | Computed (31) | Age at menopause – age at menarche | Computed |
|  | Age at last regular period | 31 | How old were you when you last had regular menstrual bleeding (a period)? (Your best guess.) | Free form entry |
|  | Ever had premenopausal hot flashes | Computed (31) | Computed from the following questions: How old were you when you first had symptoms such as hot flashes or night sweats? AND How old were you when you last had symptoms such as hot flashses or night sweats? (If you are still having symptoms…enter your current age).  If the reported age was before the reported age of menopause, we considered them to have had premenopausal hot flashes. | Computed from Free form entry responses |
|  | Ever had postmenopausal hot flashes | Computed (31) | Computed from the following questions: How old were you when you first had symptoms such as hot flashes or night sweats? AND How old were you when you last had symptoms such as hot flashses or night sweats? (If you are still having symptoms…enter your current age).  If the reported age was after the reported age of menopause, we considered them to have had postmenopausal hot flashes. | Computed from Free form entry responses |
|  | Ever had an oophorectomy | 31 | Did you ever have an operation to have one or both of your ovaries taken out? (Mark one oval.) | No; Yes, one was taken out; Yes, both were taken out; Yes, unknown number taken out; Yes, part of an ovary was taken out; Don’t know |
|  | Ever had a hysterectomy | 2 | Did you ever have a hysterectomy? (This is an operation to take out your uterus or womb.) | No; Yes |
|  | Age at last menstrual bleeding | 31 | How old were you when you last had any menstrual bleeding? | Free form entry |
|  | Age at menopause | Computed (2 & 31) | Age at which participant went through menopause. Computed using a menopause algorithm, described here: <https://www.whi.org/doc/Algorithm-Age-at-Menopause.pdf> ) | Computed |

|  | **Supplementary Table S2: Results from linear regression analyses for the associations between birthweight and age at events related to menarche and menopause, stratified by age at enrollment** | | | | | | |
| --- | --- | --- | --- | --- | --- | --- | --- |
|  |  |  | **Birth weight category**  **β (SE)**  **P** | | | |  |
|  |  |  | **< 6 lbs.** | **6 lbs. – 7 lbs. 15 oz.** | **8 lbs. – 9 lbs. 15 oz.** | **≥ 10 lbs.** | **Global P** |
| Age at first regular period | | | | | | | |
|  | 45-54 years (n cases) | | N = 882 | N = 6,226 | N = 1,797 | N = 186 |  |
|  |  | Unadjusted (N=9,091) | -0.091 (0.07)  0.19 | Ref. | -0.022 (0.05)  0.67 | -0.084 (0.14)  0.56 | 0.57 |
|  |  | Adj. for demographics (N=8,629) | -0.093 (0.07)  0.19 | Ref. | 0.001 (0.05)  0.98 | 0.025 (0.15)  0.87 | 0.61 |
|  |  | Adj. for demographic and lifestyle factors (N=7,389) | -0.072 (0.08)  0.35 | Ref. | 0.009 (0.06)  0.88 | 0.035 (0.16)  0.82 | 0.79 |
|  | 55-64 years (n cases) | | N = 2,421 | N = 18,033 | N = 5,378 | N = 802 |  |
|  |  | Unadjusted (N=26,634) | -0.055 (0.04)  0.17 | Ref. | -0.054 (0.03)  0.06 | -0.104 (0.07)  0.12 | 0.09 |
|  |  | Adj. for demographics (N=25,641) | -0.035 (0.04)  0.39 | Ref. | -0.027 (0.03)  0.35 | -0.044 (0.07)  0.52 | 0.65 |
|  |  | Adj. for demographic and lifestyle factors (N=22,534) | -0.025 (0.04)  0.57 | Ref. | -0.023 (0.03)  0.46 | -0.069 (0.07)  0.34 | 0.69 |
|  | 65-74 years (n cases) | | N = 2,156 | N = 16,729 | N = 4,975 | N = 991 |  |
|  |  | Unadjusted (N=24,851) | 0.033 (0.04)  0.41 | Ref. | -0.038 (0.03)  0.18 | -0.043 (0.06)  0.46 | 0.35 |
|  |  | Adj. for demographics (N=24,096) | 0.036 (0.04)  0.38 | Ref. | -0.012 (0.03)  0.68 | -0.009 (0.06)  0.88 | 0.77 |
|  |  | Adj. for demographic and lifestyle factors (N=21,369) | 0.039 (0.04)  0.37 | Ref. | -0.002 (0.03)  0.94 | -0.028 (0.06)  0.65 | 0.78 |
|  | ≥75 years (n cases) | | N = 439 | N = 3,120 | N = 947 | N = 207 |  |
|  |  | Unadjusted (N=4,713) | -0.012 (0.09)  0.89 | Ref. | -0.055 (0.06)  0.39 | 0.060 (0.12)  0.62 | 0.78 |
|  |  | Adj. for demographics (N=4,544) | -0.029 (0.09)  0.75 | Ref. | -0.016 (0.06)  0.81 | 0.095 (0.12)  0.44 | 0.84 |
|  |  | Adj. for demographic and lifestyle factors (N=3,982) | -0.107 (0.10)  0.26 | Ref. | -0.085 (0.07)  0.22 | 0.127 (0.13)  0.33 | 0.28 |
| Age at last regular period | | | | | | | |
|  | 45-54 years (n cases) | | N = 902 | N = 6,408 | N = 1,824 | N = 187 |  |
|  |  | Unadjusted (N=9,321) | -0.387 (0.21)  0.07 | Ref. | -0.012 (0.16)  0.94 | -0.086 (0.44)  0.84 | 0.33 |
|  |  | Adj. for demographics (N=8,866) | -0.374 (0.21)  0.08 | Ref. | 0.034 (0.16)  0.83 | 0.123 (0.45)  0.78 | 0.32 |
|  |  | Adj. for demographic and lifestyle factors (N=7,612) | -0.339 (0.23)  0.14 | Ref. | 0.082 (0.17)  0.63 | 0.426 (0.47)  0.37 | 0.30 |
|  | 55-64 years (n cases) | | N = 2,588 | N = 19,522 | N = 5,850 | N = 869 |  |
|  |  | Unadjusted (N=28,829) | -0.690 (0.14)  < 0.001 | Ref. | 0.055 (0.10)  0.59 | -0.496 (0.24)  0.03 | < 0.001 |
|  |  | Adj. for demographics (N=27,795) | -0.648 (0.14)  < 0.001 | Ref. | 0.032 (0.10)  0.76 | -0.410 (0.24)  0.08 | < 0.001 |
|  |  | Adj. for demographic and lifestyle factors (N=24,388) | -0.541 (0.15)  < 0.001 | Ref. | 0.009 (0.11)  0.93 | -0.203 (0.25)  0.42 | < 0.001 |
|  | 65-74 years (n cases) | | N = 2,355 | N = 18,139 | N = 5,418 | N = 1,109 |  |
|  |  | Unadjusted (N=27,021) | -0.492 (0.15)  < 0.001 | Ref. | 0.013 (0.10)  0.90 | -0.331 (0.20)  0.11 | 0.003 |
|  |  | Adj. for demographics (N=26,229) | -0.434 (0.15)  0.003 | Ref. | 0.020 (0.10)  0.85 | -0.298 (0.21)  0.15 | 0.01 |
|  |  | Adj. for demographic and lifestyle factors (N=23,183) | -0.376 (0.15)  0.02 | Ref. | 0.105 (0.11)  0.33 | -0.203 (0.21)  0.35 | 0.04 |
|  | ≥75 years (n cases) | | N = 475 | N = 3,367 | N = 1,019 | N = 231 |  |
|  |  | Unadjusted (N=5,092) | -0.657 (0.32) 0.04 | Ref. | 0.052 (0.24)  0.83 | 0.426 (0.45)  0.34 | 0.13 |
|  |  | Adj. for demographics (N=4,915) | -0.631 (0.33)  0.05 | Ref. | 0.105 (0.24)  0.66 | 0.411 (0.45)  0.36 | 0.15 |
|  |  | Adj. for demographic and lifestyle factors (N=4,277) | -0.529 (0.35)  0.13 | Ref. | 0.109 (0.25)  0.66 | 0.329 (0.47)  0.48 | 0.34 |
| Age at last vaginal bleeding | | | | | | | |
|  | 45-54 years (n cases) | | N = 922 | N = 6,493 | N = 1,852 | N = 194 |  |
|  |  | Unadjusted (N=9,461) | -0.30 (0.22)  0.17 | Ref. | -0.032 (0.16)  0.84 | 0.086 (0.45)  0.85 | 0.58 |
|  |  | Adj. for demographics (N=8,984) | -0.321 (0.22)  0.14 | Ref. | 0.046 (0.16)  0.78 | 0.348 (0.46)  0.45 | 0.38 |
|  |  | Adj. for demographic and lifestyle factors (N=7,690) | -0.280 (0.23)  0.23 | Ref. | 0.104 (0.17)  0.55 | 0.691 (0.48)  0.15 | 0.24 |
|  | 55-64 years (n cases) | | N = 2,664 | N = 19,873 | N = 5,958 | N = 897 |  |
|  |  | Unadjusted (N=29,392) | -0.789 (0.15)  < 0.001 | Ref. | 0.021 (0.11)  0.85 | -0.431 (0.25)  0.08 | < 0.001 |
|  |  | Adj. for demographics (N=28,325) | -0.698 (0.15)  < 0.001 | Ref. | -0.004 (0.11)  0.97 | -0.310 (0.25)  0.22 | < 0.001 |
|  |  | Adj. for demographic and lifestyle factors (N=24,854) | -0.601 (0.16)  < 0.001 | Ref. | -0.015 (0.11)  0.90 | -0.131 (0.27)  0.62 | 0.003 |
|  | 65-74 years (n cases) | | N = 2,392 | N = 18,496 | N = 5,511 | N = 1,131 |  |
|  |  | Unadjusted (N=27,530) | -0.628 (0.16)  < 0.001 | Ref. | 0.115 (0.12)  0.32 | -0.658 (0.23)  0.004 | < 0.001 |
|  |  | Adj. for demographics (N=26,726) | -0.553 (0.17)  < 0.001 | Ref. | 0.114 (0.12)  0.33 | -0.619 (0.23)  0.008 | < 0.001 |
|  |  | Adj. for demographic and lifestyle factors (N=23,615) | -0.436 (0.18)  0.01 | Ref. | 0.174 (0.12)  0.16 | -0.458 (0.24)  0.06 | 0.004 |
|  | ≥75 years (n cases) | | N = 481 | N = 3,405 | N = 1,035 | N = 234 |  |
|  |  | Unadjusted (N=5,155) | -1.279 (0.37)  < 0.001 | Ref. | -0.391 (0.27)  0.15 | 0.423 (0.52)  0.41 | 0.003 |
|  |  | Adj. for demographics (N=4,974) | -1.236 (0.38)  0.001 | Ref. | -0.353 (0.27)  0.20 | 0.338 (0.52)  0.52 | 0.007 |
|  |  | Adj. for demographic and lifestyle factors (N=4,333) | -1.052 (0.41)  0.01 | Ref. | -0.393 (0.29)  0.18 | 0.311 (0.54)  0.57 | 0.04 |
| Age at menarche | | |  |  |  |  |  |
|  | 45-54 years (n cases) | | N = 1,052 | N = 7,397 | N = 2,098 | N = 221 |  |
|  |  | Unadjusted (N=10,768) | -0.114 (0.05)  0.02 | Ref. | -0.010 (0.04)  0.79 | 0.021 (0.10)  0.84 | 0.14 |
|  |  | Adj. for demographics (N=10,206) | -0.123 (0.05)  0.01 | Ref. | 0.008 (0.04)  0.84 | 0.114 (0.10)  0.28 | 0.05 |
|  |  | Adj. for demographic and lifestyle factors (N=8,696) | -0.115 (0.05)  0.03 | Ref. | 0.003 (0.04)  0.95 | 0.094 (0.11)  0.40 | 0.14 |
|  | 55-64 years (n cases) | | N = 2,917 | N = 21,575 | N = 6,425 | N = 980 |  |
|  |  | Unadjusted (N=31,897) | -0.079 (0.03)  0.006 | Ref. | -0.021 (0.02)  0.31 | -0.007 (0.05)  0.88 | 0.0498 |
|  |  | Adj. for demographics (N=30,683) | -0.078 (0.03)  0.008 | Ref. | 0.019 (0.02)  0.36 | 0.060 (0.02)  0.36 | 0.01 |
|  |  | Adj. for demographic and lifestyle factors (N=26,859) | -0.074 (0.03)  0.02 | Ref. | 0.010 (0.02)  0.66 | 0.043 (0.05)  0.41 | 0.07 |
|  | 65-74 years (n cases) | | N = 2,579 | N = 19,830 | N = 5,914 | N = 1,211 |  |
|  |  | Unadjusted (N=29,534) | 0.033 (0.03)  0.28 | Ref. | -0.028 (0.02)  0.19) | -0.025 (0.04)  0.56 | 0.29 |
|  |  | Adj. for demographics (N=28,627) | 0.022 (0.03)  0.47 | Ref. | -0.006 (0.02)  0.78 | 0.011 (0.04)  0.79 | 0.86 |
|  |  | Adj. for demographic and lifestyle factors (N=25,263) | 0.013 (0.03)  0.70 | Ref. | -0.010 (0.020  0.67 | -0.007 (0.05)  0.88 | 0.94 |
|  | ≥75 years (n cases) | | N = 520 | N = 3,643 | N = 1,104 | N = 254 |  |
|  |  | Unadjusted (N=5,521) | -0.012 (0.07)  0.86 | Ref. | -0.054 (0.05)  0.28 | 0.088 (0.10)  0.36 | 0.52 |
|  |  | Adj. for demographics (N=5,324) | -0.025 (0.07)  0.72 | Ref. | -0.022 (0.05)  0.67 | 0.120 (0.10)  0.21 | 0.55 |
|  |  | Adj. for demographic and lifestyle factors(N=4,629) | -0.075 (0.08)  0.32 | Ref. | -0.049 (0.05)  0.36 | 0.144 (0.10)  0.15 | 0.24 |
| Reproductive window | | | | | | | |
|  | 45-54 years (n cases) | | N=667 | N=4,838 | N=1,406 | N=148 |  |
|  |  | Unadjusted (N=7,059) | 0.074 (0.20)  0.72 | Ref. | -0.203 (0.15)  0.17 | -0.468 (0.41)  0.26 | 0.34 |
|  |  | Adj. for demographic (N=6,717) | 0.774 (0.21)  0.71 | Ref. | -0.176 (0.15)  0.24 | -0.626 (0.42)  0.14 | 0.29 |
|  |  | Adj. for demographic and lifestyle factors (N=5,729) | 0.003 (0.22)  0.99 | Ref. | -0.187 (0.16)  0.24 | -0.726 (0.44)  0.10 | 0.27 |
|  | 55-64 years (n cases) | | N=1,940 | N=15,148 | N=4,429 | N=645 |  |
|  |  | Unadjusted (N=22,162) | -0.485 (0.14)  < 0.001 | Ref. | 0.133 (0.10)  0.18 | -0.413 (0.24)  0.08 | < 0.001 |
|  |  | Adj. for demographics (N=21,371) | -0.454 (0.14)  0.002 | Ref. | 0.071 (010)  0.48 | -0.467 (0.24)  0.049 | 0.002 |
|  |  | Adj. for demographic and lifestyle factors (N=18,770) | -0.317 (0.15)  0.04 | Ref. | 0.035 (0.11)  0.74 | -0.358 (0.25)  0.16 | 0.09 |
|  | 65-74 years (n cases) | | N=1,788 | N=14,167 | N=4,212 | N=850 |  |
|  |  | Unadjusted (N=21,017) | -0.471 (0.16)  0.003 | Ref. | 0.134 (0.11)  0.23 | -0.607 (0.22)  0.007 | < 0.001 |
|  |  | Adj. for demographics (N=20,395) | -0.411 (0.16)  0.01 | Ref. | 0.135 (0.11)  0.23 | -0.583 (0.23)  0.01 | 0.001 |
|  |  | Adj. for demographic and lifestyle factors (N=18,079) | -0.273 (0.17)  0.11 | Ref. | 0.162 (0.12)  0.17 | -0.295 (0.24)  0.21 | 0.07 |
|  | ≥75 years (n cases) | | N=369 | N=2,723 | N=820 | N=198 |  |
|  |  | Unadjusted (N=4,110) | -0.982 (0.35)  0.005 | Ref. | -0.311 (0.25)  0.21 | -0.031 (0.46)  0.95 | 0.03 |
|  |  | Adj. for demographics (N=3,962) | -0.908 (0.36)  0.01 | Ref. | -0.285 (0.25)  0.26 | 0.021 (0.46)  0.96 | 0.07 |
|  |  | Adj. for demographic and lifestyle factors (N=3,458) | -0.648 (0.38)  0.09 | Ref. | -0.306 (0.27)  0.25 | 0.216 (0.48)  0.66 | 0.24 |
| Age at natural menopause | | | | | | | |
|  | 45-54 years (n cases) | | N = 821 | N = 5,836 | N = 1,686 | N = 175 |  |
|  |  | Unadjusted (N=8,518) | -0.025 (0.17)  0.89 | Ref. | -0.183 (0.13)  0.16 | -0.311 (0.36)  0.39 | 0.46 |
|  |  | Adj. for demographic (N=8,097) | -0.028 (0.18)  0.87 | Ref. | -0.169 (0.13)  0.19 | -0.352 (0.37)  0.34 | 0.48 |
|  |  | Adj. for demographic and lifestyle factors (N=6,918) | -0.136 (0.19)  0.47 | Ref. | -0.179 (0.14)  0.19 | -0.416 (0.38)  0.28 | 0.41 |
|  | 55-64 years (n cases) | | N = 2,316 | N = 17,559 | N = 5,195 | N = 773 |  |
|  |  | Unadjusted (N=25,843) | -0.531 (0.13)  < 0.001 | Ref. | 0.140 (0.09)  0.12 | -0.344 (0.21)  0.10 | < 0.001 |
|  |  | Adj. for demographics (N=24,906) | -0.501 (0.13)  < 0.001 | Ref. | 0.126 (0.09)  0.16 | -0.305 (0.21)  0.15 | < 0.001 |
|  |  | Adj. for demographic and lifestyle factors (N=21,823) | -0.386 (0.14)  0.004 | Ref. | 0.106 (0.10)  0.27 | -0.159 (0.23)  0.48 | 0.01 |
|  | 65-74 years (n cases) | | N = 1,986 | N = 15,659 | N = 4,652 | N = 948 |  |
|  |  | Unadjusted (N=23,245) | -0.511 (0.15)  < 0.001 | Ref. | 0.091 (0.10)  0.38 | -0.600 (0.21)  0.004 | < 0.001 |
|  |  | Adj. for demographics (N=22,566) | -0.468 (0.15)  0.002 | Ref. | 0.111 (0.11)  0.29 | -0.522 (0.21)  0.01 | < 0.001 |
|  |  | Adj. for demographic and lifestyle factors (N=19,974) | -0.321 (0.16)  0.045 | Ref. | 0.137 (0.11)  0.22 | -0.280 (0.22)  0.21 | 0.04 |
|  | ≥75 years (n cases) | | N = 404 | N = 2,906 | N = 889 | N = 210 |  |
|  |  | Unadjusted (N=4,409) | -0.881 (0.33)  0.007 | Ref. | -0.190 (0.24)  0.42 | 0.043 (0.44)  0.92 | 0.06 |
|  |  | Adj. for demographics (N=4,253) | -0.846 (0.34)  0.01 | Ref. | -0.144 (0.24)  0.55 | 0.143 (0.44)  0.75 | 0.08 |
|  |  | Adj. for demographic and lifestyle factors (N=3,701) | -0.633 (0.36)  0.08 | Ref. | -0.162 (0.26)  0.53 | 0.408 (0.47)  0.38 | 0.23 |
| Age at natural menopause (conservative) | | | | | | | |
|  | 45-54 years (n cases) | | N = 669 | N = 4,839 | N = 1,406 | N = 148 |  |
|  |  | Unadjusted (N=7,062) | -0.010 (0.20)  0.96 | Ref. | -0.231 (0.14)  0.11 | -0.415 (0.40)  0.30 | 0.33 |
|  |  | Adj. for demographic (N=6,720) | -0.027 (0.20)  0.89 | Ref. | -0.196 (0.14)  0.17 | -0.473 (0.40)  0.24 | 0.39 |
|  |  | Adj. for demographic and lifestyle factors (N=5,730) | -0.111 (0.21)  0.60 | Ref. | -0.208 (0.15)  0.17 | -0.587 (0.42)  0.16 | 0.32 |
|  | 55-64 years (n cases) | | N = 1,941 | N = 15,164 | N = 4,432 | N = 646 |  |
|  |  | Unadjusted (N=22,183) | -0.564 (0.14)  < 0.001 | Ref. | 0.129 (0.10)  0.18 | -0.356 (0.23)  0.12 | < 0.001 |
|  |  | Adj. for demographics (N=21,390) | -0.539 (0.14)  < 0.001 | Ref. | 0.109 (0.10)  0.27 | -0.335 (0.23)  0.15 | < 0.001 |
|  |  | Adj. for demographic and lifestyle factors (N=18,786) | -0.403 (0.15)  0.006 | Ref. | 0.066 (0.10)  0.52 | -0.230 (0.25)  0.35 | 0.02 |
|  | 65-74 years (n cases) | | N = 1,790 | N = 14,192 | N = 4,216 | N = 850 |  |
|  |  | Unadjusted (N=21,048) | -0.426 (0.16)  0.006 | Ref. | 0.102 (0.11)  0.35 | -0.621 (0.22)  0.005 | < 0.001 |
|  |  | Adj. for demographics (N=20,424) | -0.371 (0.16)  0.02 | Ref. | 0.121 (0.11)  0.27 | -0.556 (0.22)  0.01 | 0.003 |
|  |  | Adj. for demographic and lifestyle factors (N=18,103) | -0.228 (0.17)  0.17 | Ref. | 0.152 (0.12)  0.19 | -0.286 (0.23)  0.22 | 0.11 |
|  | ≥75 years (n cases) | | N = 369 | N = 2,730 | N = 822 | N = 199 |  |
|  |  | Unadjusted (N=4,120) | -0.906 (0.34)  0.008 | Ref. | -0.321 (0.24)  0.19 | -0.011 (0.45)  0.98 | 0.045 |
|  |  | Adj. for demographics (N=3,972) | -0.846 (0.35)  0.01 | Ref. | -0.259 (0.25)  0.30 | 0.083 (0.45)  0.85 | 0.09 |
|  |  | Adj. for demographic and lifestyle factors (N=3,465) | -0.629 (0.37)  0.09 | Ref. | -0.306 (0.26)  0.24 | 0.289 (0.47)  0.54 | 0.22 |
| Results presented as beta (standard error) and p-value. Global P-value is testing for a linear tread. Demographic factors include age, race, ethnicity, region, and BMI. Lifestyle factors include smoking status, education, normalized socioeconomic status (NSES), and alcohol use. For the age at natural menopause analyses, participants were removed if they reported: having a bilateral oophorectomy prior to menopause; having an oophorectomy prior to menopause but did not know if it was unilateral or bilateral; or if they had an unknown oophorectomy status. For the conservative age at natural menopause, participants were also removed if they reported having a hysterectomy prior to menopause or had an unknown hysterectomy status. | | | | | | | |

|  | **Supplementary Table S3: Results from linear regression analyses for the associations between birthweight and age at events related to menarche and menopause, stratified by self-reported race and ethnicity** | | | | | | |
| --- | --- | --- | --- | --- | --- | --- | --- |
|  |  |  | **Birth weight category**  **β (SE)**  **P** | | | |  |
|  |  |  | **< 6 lbs.** | **6 lbs. – 7 lbs. 15 oz.** | **8 lbs. – 9 lbs. 15 oz.** | **≥ 10 lbs.** | **Global P** |
| Age at menarche | | |  |  |  |  |  |
|  | Asian (n cases) | | N = 347 | N = 1,349 | N = 171 | N = 21 |  |
|  |  | Unadjusted | 0.104 (0.10)  0.29 | Ref. | -0.400 (0.13)  0.003 | 0.269 (0.36)  0.45 | 0.007 |
|  |  | Adj. for demographics | 0.053 (0.10)  0.61 | Ref. | -0.213 (0.14)  0.14 | 0.536 (0.37)  0.14 | 0.17 |
|  |  | Adj. for demographic and lifestyle factors | 0.001 (0.11)  1.00 | Ref. | -0.218 (0.16)  0.16 | 0.640 (0.40)  0.11 | 0.20 |
|  | Black (n cases) | | N = 708 | N = 3,903 | N = 860 | N = 161 |  |
|  |  | Unadjusted | -0.059 (0.07)  0.38 | Ref. | -0.126 (0.06)  0.04 | 0.098 (0.13)  0.46 | 0.14 |
|  |  | Adj. for demographics | -0.055 (0.07)  0.41 | Ref. | -0.102 (0.06)  0.10 | 0.177 (0.13)  0.18 | 0.14 |
|  |  | Adj. for demographic and lifestyle factors | -0.110 (0.07)  0.14 | Ref. | -0.140 (0.07)  0.04 | 0.141 (0.15)  0.35 | 0.07 |
|  | White (n cases) | | N = 5,702 | N = 45,406 | N = 14,039 | N = 2,398 |  |
|  |  | Unadjusted | -0.058 (0.02)  0.005 | Ref. | -0.002 (0.01)  0.86 | 0.016 (0.03)  0.59 | 0.03 |
|  |  | Adj. for demographics | -0.046 (0.02)  0.02 | Ref. | 0.016 (0.01)  0.25 | 0.032 (0.03)  0.29 | 0.03 |
|  |  | Adj. for demographic and lifestyle factors | -0.045 (0.02)  0.03 | Ref. | 0.008 (0.01)  0.58 | 0.021 (0.03)  0.50 | 0.12 |
|  | Other (n cases) | | N = 114 | N = 672 | N = 197 | N = 39 |  |
|  |  | Unadjusted | -0.031 (0.17)  0.85 | Ref. | -0.219 (0.13)  0.10 | 0.210 (0.27)  0.43 | 0.30 |
|  |  | Adj. for demographics | -0.136 (0.17)  0.43 | Ref. | -0.149 (0.14)  0.28 | 0.269 (0.29)  0.35 | 0.43 |
|  |  | Adj. for demographic and lifestyle factors | -0.039 (0.19)  0.83 | Ref. | -0.124 (0.15)  0.40 | 0.150 (0.30)  0.62 | 0.77 |
|  | Hispanic/Latinx (n cases) | | N = 361 | N = 2,080 | N = 508 | N = 83 |  |
|  |  | Unadjusted | 0.142 (0.09)  0.11 | Ref. | 0.021 (0.08)  0.78 | 0.098 (0.17)  0.57 | 0.43 |
|  |  | Adj. for demographics | 0.155 (0.11)  0.18 | Ref. | 0.126 (0.10)  0.19 | 0.224 (0.21)  0.29 | 0.28 |
|  |  | Adj. for demographic and lifestyle factors | 0.126 (0.12)  0.30 | Ref. | 0.095 (0.10)  0.34 | 0.086 (0.23)  0.71 | 0.62 |
|  | Not Hispanic/Latinx (n cases) | | N = 6,594 | N = 49,904 | N = 14,927 | N = 2,563 |  |
|  |  | Unadjusted | -0.053 (0.02)  0.006 | Ref. | -0.021 (0.01)  0.12 | 0.021 (0.03)  0.48 | 0.02 |
|  |  | Adj. for demographics | -0.050 (0.02)  0.009 | Ref. | 0.002 (0.01)  0.88 | 0.042 (0.03)  0.15 | 0.02 |
|  |  | Adj. for demographic and lifestyle factors | -0.052 (0.02)  0.01 | Ref. | -0.005 (0.01)  0.71 | 0.030 (0.03)  0.33 | 0.048 |
|  | | | | | | | |
| Age at first regular period | | |  |  |  |  |  |
|  | Asian (n cases) | | N = 288 | N = 1,182 | N = 151 | N = <20 |  |
|  |  | Unadjusted | 0.006 (0.13)  0.96 | Ref. | -0.483 (0.17)  0.004 | 0.247 (0.52)  0.64 | 0.03 |
|  |  | Adj. for demographic | 0.060 (0.14)  0.66 | Ref. | -0.312 (0.19)  0.10 | 0.802 (0.55)  0.14 | 0.14 |
|  |  | Adj. for demographic and lifestyle factors | -0.017 (0.15)  0.91 | Ref. | -0.358 (0.20)  0.08 | 0.926 (0.58)  0.11 | 0.12 |
|  | Black (n cases) | | N = 574 | N = 3,216 | N = 711 | N = 134 |  |
|  |  | Unadjusted | -0.097 (0.09)  0.26 | Ref. | -0.152 (0.08)  0.05 | -0.099 (0.17)  0.55 | 0.20 |
|  |  | Adj. for demographics | -0.089 (0.09)  0.30 | Ref. | -0.123 (0.08)  0.12 | -0.017 (0.17)  0.92 | 0.38 |
|  |  | Adj. for demographic and lifestyle factors | -0.142 (0.10)  0.14 | Ref. | -0.196 (0.09)  0.02 | -0.053 (0.19)  0.78 | 0.10 |
|  | White (n cases) | | N = 4,788 | N = 38,320 | N = 11,834 | N = 1,977 |  |
|  |  | Unadjusted | -0.026 (0.03)  0.35 | Ref. | -0.023 (0.02)  0.24 | -0.049 (0.04)  0.24 | 0.40 |
|  |  | Adj. for demographics | -0.014 (0.03)  0.62 | Ref. | -0.003 (0.02)  0.88 | -0.014 (0.04)  0.74 | 0.95 |
|  |  | Adj. for demographic and lifestyle factors | -0.007 (0.03)  0.82 | Ref. | 0.01 (0.02)  0.96 | -0.027 (0.04)  0.54 | 0.94 |
| Other (n cases) | | | N = 94 | N = 569 | N = 171 | N = 27 |  |
|  |  | Unadjusted | 0.039 (0.22)  0.86 | Ref. | -0.316 (0.18)  0.07 | 0.021 (0.40)  0.96 | 0.31 |
|  |  | Adj. for demographics | 0.034 (0.24)  0.89 | Ref. | -0.269 (0.18)  0.14 | 0.076 (0.42)  0.86 | 0.49 |
|  |  | Adj. for demographic and lifestyle factors | 0.327 (0.25)  0.20 | Ref. | -0.204 (0.19)  0.29 | 0.095 (0.43)  0.82 | 0.34 |
| Hispanic/Latinx (n cases) | | | N = 285 | N = 1,717 | N = 425 | N = 68 |  |
|  |  | Unadjusted | 0.055 (0.12)  0.65 | Ref. | -0.039 (0.10)  0.70 | 0.135 (0.23)  0.57 | 0.86 |
|  |  | Adj. for demographics | 0.055 (0.16)  0.73 | Ref. | 0.039 (0.13)  0.77 | 0.185 (0.29)  0.53 | 0.91 |
|  |  | Adj. for demographic and lifestyle factors | 0.090 (0.17)  0.60 | Ref. | 0.019 (0.14)  0.89 | 0.118 (0.32)  0.71 | 0.94 |
| Not Hispanic/Latinx (n cases) | | | N = 5,524 | N = 41,995 | N = 12,579 | N = 2,106 |  |
|  |  | Unadjusted | -0.026 (0.03)  0.31 | Ref. | -0.040 (0.02)  0.03 | -0.052 (0.04)  0.20 | 0.11 |
|  |  | Adj. for demographics | -0.018 (0.03)  0.50 | Ref. | -0.018 (0.02)  0.33 | -0.015 (0.04)  0.71 | 0.73 |
|  |  | Adj. for demographic and lifestyle factors | -0.014 (0.03)  0.61 | Ref. | -0.016 (0.02)  0.40 | -0.029 (0.04)  0.51 | 0.76 |
|  | | | | | | | |
| Age at last regular period | | |  |  |  |  |  |
|  | Asian (n cases) | | N = 314 | N = 1,221 | N = 162 | N = <20 |  |
|  |  | Unadjusted | -0.359 (0.39)  0.36 | Ref. | 0.555 (0.52)  0.28 | -1.564 (1.43)  0.27 | 0.32 |
|  |  | Adj. for demographics | -0.440 (0.43)  0.31 | Ref. | 0.765 (0.58)  0.19 | -2.028 (1.54)  0.19 | 0.18 |
|  |  | Adj. for demographic and lifestyle factors | -0.525 (0.46)  0.25 | Ref. | 0.947 (0.64)  0.14 | -0.580 (1.72)  0.74 | 0.24 |
|  | Black (n cases) | | N = 611 | N = 3,379 | N = 754 | N = 134 |  |
|  |  | Unadjusted | -0.256 (0.32)  0.43 | Ref. | 0.254 (0.30)  0.39 | 0.254 (0.65)  0.69 | 0.62 |
|  |  | Adj. for demographics | -0.319 (0.32)  0.32 | Ref. | 0.257 (0.30)  0.39 | 0.247 (0.65)  0.70 | 0.53 |
|  |  | Adj. for demographic and lifestyle factors | -0.354 (0.36)  0.33 | Ref. | 0.358 (0.33)  0.27 | -0.090 (0.75)  0.90 | 0.46 |
|  | White (n cases) | | N = 5,144 | N = 41,417 | N = 12,805 | N = 2,166 |  |
|  |  | Unadjusted | -0.560 (0.10)  < 0.001 | Ref. | -0.036 (0.07)  0.58 | -0.170 (0.14)  0.24 | < 0.001 |
|  |  | Adj. for demographics | -0.536 (0.10)  < 0.001 | Ref. | 0.004 (0.07)  0.95 | -0.244 (0.14)  0.09 | < 0.001 |
|  |  | Adj. for demographic and lifestyle factors | -0.432 (0.10)  < 0.001 | Ref. | 0.025 (0.07)  0.72 | -0.088 (0.15)  0.56 | < 0.001 |
|  | Other( n cases) | | N = 94 | N = 582 | N = 175 | N = 34 |  |
|  |  | Unadjusted | -1.006 (0.767)  0.19 | Ref. | 0.639 (0.59)  0.28 | 0.872 (1.22)  0.47 | 0.26 |
|  |  | Adj. for demographics | -1.312 (0.79)  0.10 | Ref. | 0.627 (0.61)  0.30 | 0.87 (1.29)  0.50 | 0.18 |
|  |  | Adj. for demographic and lifestyle factors | -1.268 (0.86)  0.14 | Ref. | 1.143 (0.65)  0.08 | 1.152 (1.35)  0.39 | 0.08 |
|  | Hispanic/Latinx (n cases) | | N = 302 | N = 1,763 | N = 428 | N = 71 |  |
|  |  | Unadjusted | 0.216 (0.44)  0.62 | Ref. | 0.154 (0.38)  0.69 | 1.018 (0.85)  0.23 | 0.65 |
|  |  | Adj. for demographics | 0.120 (0.56)  0.83 | Ref. | 0.017 (0.46)  0.97 | 2.097 (1.06)  0.048 | 0.27 |
|  |  | Adj. for demographic and lifestyle factors | 0.373 (0.60)  0.53 | Ref. | -0.071 (0.49)  0.89 | 2.200 (1.13)  0.051 | 0.24 |
|  | Not Hispanic/Latinx (n cases) | | N = 5,919 | N = 45,272 | N = 13,594 | N = 2,306 |  |
|  |  | Unadjusted | -0.618 (0.09)  < 0.001 | Ref. | 0.029 (0.06)  0.66 | -0.169 (0.14)  0.23 | < 0.001 |
|  |  | Adj. for demographics | -0.546 (0.09)  < 0.001 | Ref. | 0.034 (0.06)  0.60 | -0.262 (0.14)  0.06 | < 0.001 |
|  |  | Adj. for demographic and lifestyle factors | -0.471 (0.10)  < 0.001 | Ref. | 0.064 (0.07)  0.34 | -0.127 (0.15)  0.39 | < 0.001 |
|  | | | | | | | |
| Age at last bleeding | | |  |  |  |  |  |
|  | Asian (n cases) | | N = 321 | N = 1,264 | N = 166 | N = <20 |  |
|  |  | Unadjusted | -0.571 (0.41)  0.17 | Ref. | 0.895 (0.55)  0.10 | -2.744 (1.53)  0.07 | 0.04 |
|  |  | Adj. for demographics | -0.777 (0.46)  0.09 | Ref. | 1.059 (0.62)  0.09 | -3.613 (1.65)  0.03 | 0.009 |
|  |  | Adj. for demographic and lifestyle factors | -0.835 (0.49)  0.09 | Ref. | 1.012 (0.67)  0.13 | -2.215 (1.86)  0.23 | 0.06 |
|  | Black (n cases) | | N = 636 | N = 3,493 | N = 762 | N = 144 |  |
|  |  | Unadjusted | -0.081 (0.33)  0.81 | Ref. | 0.167 (0.31)  0.59 | -0.239 (0.65)  0.71 | 0.91 |
|  |  | Adj. for demographics | -0.142 (0.33)  0.67 | Ref. | 0.169 (0.31)  0.58 | -0.260 (0.66)  0.69 | 0.86 |
|  |  | Adj. for demographic and lifestyle factors | -0.171 (0.38)  0.65 | Ref. | 0.265 (0.34)  0.44 | -0.694 (0.76)  0.36 | 0.61 |
|  | White (n cases) | | N = 5,234 | N = 42,043 | N = 13,021 | N = 2,214 |  |
|  |  | Unadjusted | -0.688 (0.11)  < 0.001 | Ref. | -0.043 (0.07)  0.55 | -0.226 (0.16)  0.15 | < 0.001 |
|  |  | Adj. for demographics | -0.651 (0.11)  < 0.001 | Ref. | 0.003 (0.07)  0.97 | -0.326 (0.16)  0.04 | < 0.001 |
|  |  | Adj. for demographic and lifestyle factors | -0.515 (0.11)  < 0.001 | Ref. | 0.017 (0.08)  0.82 | -0.132 (0.17)  0.43 | < 0.001 |
|  | Other (n cases) | | N = 99 | N = 607 | N = 181 | N = 38 |  |
|  |  | Unadjusted | -1.578 (0.80)  0.0495 | Ref. | -0.127 (0.63)  0.84 | 1.375 (1.24)  0.27 | 0.14 |
|  |  | Adj. for demographics | -2.080 (0.85)  0.01 | Ref. | 0.025 (0.65)  0.97 | 1.804 (1.33)  0.18 | 0.04 |
|  |  | Adj. for demographic and lifestyle factors | -2.190 (0.92)  0.02 | Ref. | 0.694 (0.69)  0.31 | 1.726 (1.35)  0.20 | 0.03 |
|  | Hispanic/Latinx (n cases) | | N = 315 | N = 1,817 | N = 440 | N = 74 |  |
|  |  | Unadjusted | -0.076 (0.46)  0.87 | Ref. | -0.113 (0.40)  0.78 | 0.520 (0.89)  0.56 | 0.92 |
|  |  | Adj. for demographics | -0.129 (0.60)  0.83 | Ref. | -0.037 (0.49)  0.94 | 1.773 (1.09)  0.10 | 0.42 |
|  |  | Adj. for demographic and lifestyle factors | 0.126 (0.63)  0.84 | Ref. | -0.103 (0.52)  0.84 | 1.661 (1.15)  0.15 | 0.53 |
|  | Not Hispanic/Latinx (n cases) | | N = 6,045 | N = 46,037 | N = 13,822 | N = 2,363 |  |
|  |  | Unadjusted | -0.738 (0.10)  < 0.001 | Ref. | 0.027 (0.07)  0.71 | -0.244 (0.15)  0.11 | < 0.001 |
|  |  | Adj. for demographics | -0.645 (0.10)  < 0.001 | Ref. | 0.026 (0.07)  0.72 | -0.355 (0.15)  0.02 | < 0.001 |
|  |  | Adj. for demographic and lifestyle factors | -0.546 (0.11)  < 0.001 | Ref. | 0.049 (0.07)  0.51 | -0.190 (0.16)  0.24 | < 0.001 |
|  | | | | | | | |
| Age at natural menopause | | |  |  |  |  |  |
|  | Asian/PI (n cases) | | N = 279 | N = 1,086 | N = 131 | N = <20 |  |
|  |  | Unadjusted | -0.616 (0.36)  0.09 | Ref. | 0.819 (0.50)  0.10 | -0.931 (1.45)  0.52 | 0.07 |
|  |  | Adj. for demographics | -0.588 (0.40)  0.15 | Ref. | 0.964 (0.57)  0.09 | -0.945 (1.61)  0.56 | 0.11 |
|  |  | Adj. for demographic and lifestyle factors | -0.531 (0.43)  0.22 | Ref. | 1.123 (0.64)  0.08 | 0.176 (1.82)  0.92 | 0.15 |
|  | Black (n cases) | | N = 504 | N = 2,746 | N = 603 | N = 112 |  |
|  |  | Unadjusted | 0.038 (0.34)  0.91 | Ref. | 0.122 (0.31)  0.70 | -0.617 (0.67)  0.36 | 0.78 |
|  |  | Adj. for demographics | 0.040 (0.34)  0.91 | Ref. | 0.168 (0.32)  0.60 | -0.582 (0.68)  0.39 | 0.77 |
|  |  | Adj. for demographic and lifestyle factors | 0.147 (0.39)  0.70 | Ref. | 0.289 (0.35)  0.41 | -0.673 (0.79)  0.40 | 0.67 |
|  | White (n cases) | | N = 4,518 | N = 36,882 | N = 11,345 | N = 1,914 |  |
|  |  | Unadjusted | -0.487 (0.09)  < 0.001 | Ref. | 0.030 (0.06)  0.63 | -0.299 (0.13)  0.03 | < 0.001 |
|  |  | Adj. for demographics | -0.476 (0.09)  < 0.001 | Ref. | 0.047 (0.06)  0.45 | -0.360 (0.14)  0.008 | < 0.001 |
|  |  | Adj. for demographic and lifestyle factors | -0.365 (0.10)  < 0.001 | Ref. | 0.038 (0.06)  0.56 | -0.175 (0.14)  0.22 | < 0.001 |
|  | Other (n cases) | | N = 76 | N = 506 | N = 149 | N = 31 |  |
|  |  | Unadjusted | -1.313 (0.74)  0.08 | Ref. | -0.416 (0.56)  0.46 | 2.155 (1.11)  0.05 | 0.047 |
|  |  | Adj. for demographics | -1.356 (0.77)  0.08 | Ref. | -0.118 (0.59)  0.84 | 2.512 (1.21)  0.04 | 0.046 |
|  |  | Adj. for demographic and lifestyle factors | -1.686 (0.85)  0.048 | Ref. | -0.025 (0.64)  0.97 | 2.721 (1.28)  0.03 | 0.03 |
|  | Hispanic/Latinx (n cases) | | N = 277 | N = 1,585 | N = 387 | N = 63 |  |
|  |  | Unadjusted | -0.283 (0.40)  0.47 | Ref. | 0.280 (0.34)  0.42 | 0.571 (0.78)  0.46 | 0.59 |
|  |  | Adj. for demographics | -0.329 (0.52)  0.53 | Ref. | -0.119 (0.42)  0.78 | 1.394 (0.98)  0.15 | 0.45 |
|  |  | Adj. for demographic and lifestyle factors | -0.268 (0.55)  0.63 | Ref. | -0.131 (0.45)  0.77 | 1.112 (1.08)  0.30 | 0.69 |
|  | Not Hispanic/Latinx (n cases) | | N = 5,165 | N = 40,037 | N = 11,955 | N = 2,028 |  |
|  |  | Unadjusted | -0.488 (0.09)  < 0.001 | Ref. | 0.048 (0.06)  0.43 | -0.326 (0.13)  0.01 | < 0.001 |
|  |  | Adj. for demographics | -0.453 (0.09)  < 0.001 | Ref. | 0.063 (0.06)  0.30 | -0.374 (0.13)  0.005 | < 0.001 |
|  |  | Adj. for demographic and lifestyle factors | -0.356 (0.09)  < 0.001 | Ref. | 0.063 (0.06)  0.33 | -0.180 (0.14)  0.20 | < 0.001 |
|  | | | | | | | |
| Age at natural menopause (conservative) | | |  |  |  |  |  |
|  | Asian/PI (n cases) | | N = 256 | N = 1,002 | N = 121 | N = <20 |  |
|  |  | Unadjusted | -0.561 (0.37)  0.13 | Ref. | 0.940 (0.51)  0.06 | -1.480 (1.47)  0.32 | 0.053 |
|  |  | Adj. for demographics | -0.469 (0.41)  0.26 | Ref. | 0.913 (0.58)  0.11 | -1.680 (1.65)  0.31 | 0.15 |
|  |  | Adj. for demographic and lifestyle factors | -0.410 (0.44)  0.35 | Ref. | 1.052 (0.64)  0.10 | -0.586 (1.88)  0.76 | 0.25 |
|  | Black (n cases) | | N = 385 | N = 2,168 | N = 487 | N = 90 |  |
|  |  | Unadjusted | -0.063 (0.40)  0.87 | Ref. | -0.020 (0.36)  0.95 | -0.969 (0.77)  0.21 | 0.66 |
|  |  | Adj. for demographics | -0.116 (0.40)  0.77 | Ref. | -0.020 (0.36)  0.96 | -0.983 (0.78)  0.21 | 0.65 |
|  |  | Adj. for demographic and lifestyle factors | 0.031 (0.46)  0.95 | Ref. | 0.061 (0.40)  0.88 | -1.039 (0.92)  0.26 | 0.72 |
|  | White (n cases) | | N = 3,931 | N = 32,690 | N = 9,972 | N = 1,685 |  |
|  |  | Unadjusted | -0.460 (0.10)  < 0.001 | Ref. | 0.015 (0.07)  0.82 | -0.356 (0.14)  0.01 | < 0.001 |
|  |  | Adj. for demographics | -0.446 (0.10)  < 0.001 | Ref. | 0.037 (0.07)  0.57 | -0.393 (0.14)  0.01 | < 0.001 |
|  |  | Adj. for demographic and lifestyle factors | -0.325 (0.10)  0.002 | Ref. | 0.020 (0.07)  0.77 | -0.228 (0.15)  0.13 | 0.006 |
|  | Other (n cases) | | N = 65 | N = 429 | N = 132 | N = 27 |  |
|  |  | Unadjusted | -1.172 (0.81)  0.15 | Ref. | -0.340 (0.61)  0.57 | 2.394 (1.21)  0.048 | 0.08 |
|  |  | Adj. for demographics | -1.183 (0.83)  0.16 | Ref. | 0.029 (0.64)  0.96 | 2.892 (1.30)  0.03 | 0.06 |
|  |  | Adj. for demographic and lifestyle factors | -1.446 (0.93)  0.12 | Ref. | 0.227 (0.68)  0.74 | 3.414 (1.37)  0.01 | 0.03 |
|  | Hispanic/Latinx (n cases) | | N = 243 | N = 1,321 | N = 317 | N = 50 |  |
|  |  | Unadjusted | -0.186 (0.42)  0.66 | Ref. | 0.342 (0.38)  0.37 | 1.198 (0.87)  0.17 | 0.40 |
|  |  | Adj. for demographics | -0.103 (0.56)  0.85 | Ref. | -0.100 (0.47)  0.83 | 1.981 (1.08)  0.07 | 0.31 |
|  |  | Adj. for demographic and lifestyle factors | -0.072 (0.59)  0.90 | Ref. | 0.041 (0.50)  0.93 | 1.597 (1.19)  0.18 | 0.61 |
| Not Hispanic/Latinx (n cases) | | | N = 4,448 | N = 35,307 | N = 10,488 | N = 1,779 |  |
|  |  | Unadjusted | -0.469 (0.09)  < 0.001 | Ref. | 0.024 (0.06)  0.71 | -0.408 (0.14)  0.004 | < 0.001 |
|  |  | Adj. for demographics | -0.441 (0.09)  < 0.001 | Ref. | 0.045 (0.07)  0.49 | -0.433 (0.14)  0.002 | < 0.001 |
|  |  | Adj. for demographic and lifestyle factors | -0.329 (0.10)  < 0.001 | Ref. | 0.035 (0.07)  0.61 | -0.248 (0.15)  0.10 | 0.002 |
|  | | | | | | | |
| Reproductive window | | |  |  |  |  |  |
|  | Asian/PI (n cases) | | N = 256 | N = 1,002 | N = 121 | N = <20 |  |
|  |  | Unadjusted | -0.692 (0.39)  0.07 | Ref. | 1.289 (0.53)  0.02 | -1.558 (1.54)  0.31 | 0.009 |
|  |  | Adj. for demographics | -0.546 (0.43)  0.21 | Ref. | 1.064 (0.61)  0.08 | -2.126 (1.74)  0.22 | 0.08 |
|  |  | Adj. for demographic and lifestyle factors | -0.445 (0.47)  0.34 | Ref. | 1.220 (0.68)  0.07 | -1.292 (1.99)  0.52 | 0.17 |
|  | Black (n cases) | | N = 384 | N = 2,164 | N = 485 | N = 90 |  |
|  |  | Unadjusted | 0.074 (0.40)  0.85 | Ref. | 0.133 (0.36)  0.72 | -0.926 (0.78)  0.24 | 0.64 |
|  |  | Adj. for demographics | 0.019 (0.41)  0.96 | Ref. | 0.130 (0.37)  0.72 | -1.033 (0.79)  0.19 | 0.59 |
|  |  | Adj. for demographic and lifestyle factors | 0.264 (0.46)  0.57 | Ref. | 0.197 (0.41)  0.63 | -1.115 (0.94)  0.23 | 0.55 |
|  | White (n cases) | | N = 3,927 | N = 32,647 | N = 9,965 | N = 1,683 |  |
|  |  | Unadjusted | -0.427 (0.10)  < 0.001 | Ref. | 0.010 (0.07)  0.89 | -0.412 (0.15)  0.005 | < 0.001 |
|  |  | Adj. for demographics | -0.422 (0.10)  < 0.001 | Ref. | 0.014 (0.07)  0.83 | -0.465 (0.15)  0.002 | < 0.001 |
|  |  | Adj. for demographic and lifestyle factors | -0.305 (0.10)  0.004 | Ref. | 0.004 (0.07)  0.96 | -0.287 (0.16)  0.06 | 0.009 |
|  | Other (n cases) | | N = 65 | N = 429 | N = 132 | N = 27 |  |
|  |  | Unadjusted | -1.066 (0.82)  0.20 | Ref. | -0.029 (0.62)  0.96 | 2.232 (1.23)  0.07 | 0.14 |
|  |  | Adj. for demographics | -0.969 (0.85)  0.26 | Ref. | 0.273 (0.65)  0.68 | 2.589 (1.33)  0.053 | 0.13 |
|  |  | Adj. for demographic and lifestyle factors | -1.373 (0.94)  0.14 | Ref. | 0.428 (0.69)  0.54 | 3.169 (1.39)  0.02 | 0.04 |
|  | Hispanic/Latinx (n cases) | | N = 243 | N = 1,319 | N = 316 | N = 50 |  |
|  |  | Unadjusted | -0.286 (0.43)  0.51 | Ref. | 0.332 (0.39)  0.39 | 0.779 (0.89)  0.38 | 0.54 |
|  |  | Adj. for demographics | -0.186 (0.57)  0.74 | Ref. | -0.209 (0.48)  0.66 | 1.247 (1.10)  0.26 | 0.64 |
|  |  | Adj. for demographic and lifestyle factors | -0.049 (0.60)  0.93 | Ref. | -0.044 (0.51)  0.93 | 0.969 (1.22)  0.43 | 0.88 |
|  | Not Hispanic/Latinx (n cases) | | N = 4,443 | N = 35,260 | N = 10,480 | N = 1,777 |  |
|  |  | Unadjusted | -0.435 (0.10)  < 0.001 | Ref. | 0.040 (0.07)  0.55 | -0.448 (0.15)  0.002 | < 0.001 |
|  |  | Adj. for demographics | -0.409 (0.10)  < 0.001 | Ref. | 0.038 (0.07)  0.57 | -0.495 (0.15)  < 0.001 | < 0.001 |
|  |  | Adj. for demographic and lifestyle factors | -0.298 (0.10)  0.003 | Ref. | 0.031 (0.07)  0.66 | -0.302 (0.15)  0.049 | 0.005 |
| Results presented as beta (standard error) and p-value. Global P-value is testing for a linear tread. Demographic factors include age, race, ethnicity, region, and BMI. Lifestyle factors include smoking status, education, normalized socioeconomic status (NSES), and alcohol use. For the age at natural menopause analyses, participants were removed if they reported: having a bilateral oophorectomy prior to menopause; having an oophorectomy prior to menopause but did not know if it was unilateral or bilateral; or if they had an unknown oophorectomy status. For the conservative age at natural menopause, participants were also removed if they reported having a hysterectomy prior to menopause or had an unknown hysterectomy status. | | | | | | | |

|  | **Supplementary Table S4: Results from logistic regression analyses for the associations between birthweight and age at events related to menarche and menopause, stratified by age at enrollment** | | | | | | |
| --- | --- | --- | --- | --- | --- | --- | --- |
|  |  |  | **Birth weight category**  **OR (95% CI)** | | | |  |
|  |  |  | **< 6 lbs.** | **6 lbs. – 7 lbs. 15 oz.** | **8 lbs. – 9 lbs. 15 oz.** | **≥ 10 lbs.** | **Global P** |
| Were periods regular | | |  |  |  |  |  |
|  | 45-54 years (n cases) | | 838 | 6,023 | 1,706 | 174 |  |
|  |  | Unadjusted | 0.89 (0.76-1.05) | 1.00 (Ref.) | 0.99 (0.87-1.12) | 0.84 (0.61-1.17) | 0.41 |
|  |  | Adj. for demographics | 0.88 (0.74-1.04) | 1.00 (Ref.) | 0.98 (0.86-1.12) | 0.83 (0.59-1.17) | 0.36 |
|  |  | Adj. for demographic and lifestyle factors | 0.85 (0.71-1.01) | 1.00 (Ref.) | 0.97 (0.85-1.12) | 0.80 (0.56-1.14) | 0.21 |
|  | 55-64 years (n cases) | | 2,307 | 17,643 | 5,198 | 772 |  |
|  |  | Unadjusted | 0.85 (0.77-0.93) | 1.00 (Ref.) | 0.95 (0.89-1.02) | 0.83 (0.71-0.97) | 0.001 |
|  |  | Adj. for demographics | 0.84 (0.76-0.93) | 1.00 (Ref.) | 0.96 (0.90-1.04) | 0.85 (0.73-1.00) | 0.002 |
|  |  | Adj. for demographic and lifestyle factors | 0.84 (0.76-0.94) | 1.00 (Ref.) | 0.97 (0.89-1.04) | 0.86 (0.72-1.03) | 0.006 |
|  | 65-74 years (n cases) | | 2,094 | 16,602 | 4,917 | 984 |  |
|  |  | Unadjusted | 0.85 (0.77-0.95) | 1.00 (Ref.) | 0.96 (0.89-1.04) | 0.86 (0.74-1.00) | 0.009 |
|  |  | Adj. for demographics | 0.84 (0.76-0.94) | 1.00 (Ref.) | 0.97 (0.89-1.05) | 0.87 (0.75-1.02) | 0.009 |
|  |  | Adj. for demographic and lifestyle factors | 0.85 (0.76-0.96) | 1.00 (Ref.) | 0.97 (0.90-1.06) | 0.88 (0.75-1.03) | 0.03 |
|  | ≥75 years (n cases) | | 424 | 3,127 | 942 | 209 |  |
|  |  | Unadjusted | 0.72 (0.57-0.92) | 1.00 (Ref.) | 0.96 (0.79-1.16) | 0.77 (0.55-1.08) | 0.03 |
|  |  | Adj. for demographics | 0.74 (0.57-0.95) | 1.00 (Ref.) | 0.96 (0.79-1.17) | 0.76 (0.54-1.07) | 0.05 |
|  |  | Adj. for demographic and lifestyle factors | 0.74 (0.56-0.96) | 1.00 (Ref.) | 0.95 (0.77-1.16) | 0.87 (0.60-1.26) | 0.15 |
|  | | | | | | | |
| Ever had premenopausal hot flashes | | |  |  |  |  |  |
|  | 45-54 years (n cases) | | 871 | 6,175 | 1,765 | 188 |  |
|  |  | Unadjusted | 0.95 (0.80-1.12) | 1.00 (Ref.) | 1.06 (0.93-1.21) | 1.15 (0.79-1.67) | 0.59 |
|  |  | Adj. for demographics | 0.94 (0.79-1.13) | 1.00 (Ref.) | 1.06 (0.92-1.21) | 1.09 (0.75-1.60) | 0.69 |
|  |  | Adj. for demographic and lifestyle factors | 0.94 (0.78-1.14) | 1.00 (Ref.) | 0.98 (0.85-1.14) | 1.05 (0.70-1.56) | 0.93 |
|  | 55-64 years (n cases) | | 2,379 | 17,827 | 5,331 | 810 |  |
|  |  | Unadjusted | 0.93 (0.84-1.02) | 1.00 (Ref.) | 1.03 (0.95-1.11) | 0.99 (0.84-1.17) | 0.35 |
|  |  | Adj. for demographics | 0.93 (0.84-1.03) | 1.00 (Ref.) | 1.01 (0.94-1.09) | 0.96 (0.81-1.14) | 0.47 |
|  |  | Adj. for demographic and lifestyle factors | 0.93 (0.83-1.04) | 1.00 (Ref.) | 1.01 (0.93-1.09) | 1.01 (0.84-1.22) | 0.62 |
|  | 65-74 years (n cases) | | 2,157 | 16,747 | 5,020 | 1,024 |  |
|  |  | Unadjusted | 0.95 (0.85-1.06) | 1.00 (Ref.) | 1.04 (0.96-1.13) | 1.02 (0.87-1.19) | 0.49 |
|  |  | Adj. for demographics | 0.96 (0.86-1.07) | 1.00 (Ref.) | 1.05 (0.96-1.13) | 1.00 (0.85-1.17) | 0.59 |
|  |  | Adj. for demographic and lifestyle factors | 1.00 (0.89-1.13) | 1.00 (Ref.) | 1.05 (0.96-1.15) | 1.06 (0.89-1.26) | 0.68 |
|  | ≥75 years (n cases) | | 450 | 3,153 | 948 | 220 |  |
|  |  | Unadjusted | 1.02 (0.78-1.33) | 1.00 (Ref.) | 0.96 (0.80-1.17) | 0.97 (0.67-1.39) | 0.98 |
|  |  | Adj. for demographics | 1.02 (0.78-1.34) | 1.00 (Ref.) | 0.99 (0.82-1.21) | 0.95 (0.66-1.37) | 0.99 |
|  |  | Adj. for demographic and lifestyle factors | 0.93 (0.69-1.24) | 1.00 (Ref.) | 1.02 (0.82-1.26) | 0.97 (0.66-1.44) | 0.95 |
|  |  |  |  |  |  |  |  |
| Ever had postmenopausal hot flashes | | | | | | | |
|  | 45-54 years (n cases) | | 375 | 2,501 | 725 | 65 |  |
|  |  | Unadjusted | 1.05 (0.89-1.24) | 1.00 (Ref.) | 0.99 (0.88-1.13) | 0.86 (0.60-1.23) | 0.78 |
|  |  | Adj. for demographics | 1.06 (0.89-1.27) | 1.00 (Ref.) | 0.98 (0.86-1.12) | 0.89 (0.60-1.31) | 0.80 |
|  |  | Adj. for demographic and lifestyle factors | 1.11 (0.92-1.35) | 1.00 (Ref.) | 1.04 (0.90-1.19) | 0.91 (0.60-1.38) | 0.68 |
|  | 55-64 years (n cases) | | 1,010 | 7,595 | 2,268 | 355 |  |
|  |  | Unadjusted | 0.97 (0.88-1.07) | 1.00 (Ref.) | 0.99 (0.93-1.06) | 1.03 (0.88-1.20) | 0.90 |
|  |  | Adj. for demographics | 0.97 (0.88-1.07) | 1.00 (Ref.) | 1.00 (0.93-1.07) | 1.04 (0.89-1.23) | 0.89 |
|  |  | Adj. for demographic and lifestyle factors | 0.97 (0.88-1.08) | 1.00 (Ref.) | 1.01 (0.93-1.08) | 1.01 (0.85-1.20) | 0.96 |
|  | 65-74 years (n cases) | | 816 | 6,366 | 1,829 | 393 |  |
|  |  | Unadjusted | 0.99 (0.90-1.09) | 1.00 (Ref.) | 0.95 (0.89-1.02) | 1.02 (0.89-1.17) | 0.50 |
|  |  | Adj. for demographics | 1.01 (0.92-1.12) | 1.00 (Ref.) | 0.94 (0.87-1.01) | 1.01 (0.88-1.16) | 0.30 |
|  |  | Adj. for demographic and lifestyle factors | 0.99 (0.89-1.11) | 1.00 (Ref.) | 0.93 (0.87-1.01) | 0.99 (0.86-1.15) | 0.35 |
|  | ≥75 years (n cases) | | 154 | 1,069 | 349 | 69 |  |
|  |  | Unadjusted | 1.01 (0.81-1.26) | 1.00 (Ref.) | 1.15 (0.98-1.34) | 0.86 (0.63-1.16) | 0.23 |
|  |  | Adj. for demographics | 1.03 (0.82-1.28) | 1.00 (Ref.) | 1.11 (0.94-1.30) | 0.85 (0.62-1.15) | 0.39 |
|  |  | Adj. for demographic and lifestyle factors | 1.06 (0.84-1.35) | 1.00 (Ref.) | 1.10 (0.92-1.30) | 0.85 (0.62-1.18) | 0.49 |
| Ever had an oophorectomy | | | | | | | |
|  | 45-54 years (n cases) | | 309 | 2,057 | 554 | 65 |  |
|  |  | Unadjusted | 1.08 (0.94-1.24) | 1.00 (Ref.) | 0.93 (0.83-1.04) | 1.08 (0.81-1.45) | 0.30 |
|  |  | Adj. for demographics | 1.09 (0.94-1.27) | 1.00 (Ref.) | 0.92 (0.82-1.03) | 1.06 (0.78-1.44) | 0.25 |
|  |  | Adj. for demographic and lifestyle factors | 1.07 (0.91-1.25) | 1.00 (Ref.) | 0.90 (0.80-1.02) | 0.99 (0.72-1.38) | 0.28 |
|  | 55-64 years (n cases) | | 887 | 5,760 | 1,796 | 292 |  |
|  |  | Unadjusted | 1.20 (1.10-1.31) | 1.00 (Ref.) | 1.07 (1.00-1.13) | 1.16 (1.01-1.34) | < 0.001 |
|  |  | Adj. for demographics | 1.20 (1.10-1.31) | 1.00 (Ref.) | 1.05 (0.99-1.12) | 1.12 (0.97-1.29) | < 0.001 |
|  |  | Adj. for demographic and lifestyle factors | 1.20 (1.09-1.32) | 1.00 (Ref.) | 1.06 (0.99-1.14) | 1.12 (0.96-1.31) | < 0.001 |
|  | 65-74 years (n cases) | | 821 | 5,996 | 1,832 | 414 |  |
|  |  | Unadjusted | 1.08 (0.99-1.19) | 1.00 (Ref.) | 1.04 (0.97-1.10) | 1.20 (1.06-1.36) | 0.01 |
|  |  | Adj. for demographics | 1.08 (0.99-1.18) | 1.00 (Ref.) | 1.03 (0.96-1.10) | 1.18 (1.04-1.34) | 0.03 |
|  |  | Adj. for demographic and lifestyle factors | 1.08 (0.98-1.19) | 1.00 (Ref.) | 1.02 (0.96-1.10) | 1.21 (1.06-1.38) | 0.02 |
|  | ≥75 years (n cases) | | 183 | 1,108 | 346 | 77 |  |
|  |  | Unadjusted | 1.27 (1.04-1.54) | 1.00 (Ref.) | 1.04 (0.90-1.20) | 1.02 (0.77-1.34) | 0.13 |
|  |  | Adj. for demographics | 1.32 (1.08-1.61) | 1.00 (Ref.) | 1.03 (0.89-1.20) | 0.98 (0.74-1.31) | 0.06 |
|  |  | Adj. for demographic and lifestyle factors | 1.30 (1.05-1.62) | 1.00 (Ref.) | 1.06 (0.90-1.24) | 0.92 (0.68-1.25) | 0.09 |
|  |  |  |  |  |  |  |  |
| Ever had a hysterectomy | | | | | | | |
|  | 45-54 years (n cases) | | 445 | 2,988 | 829 | 84 |  |
|  |  | Unadjusted | 1.08 (0.95-1.23) | 1.00 (Ref.) | 0.97 (0.88-1.07) | 0.91 (0.69-1.20) | 0.45 |
|  |  | Adj. for demographics | 1.09 (0.95-1.25) | 1.00 (Ref.) | 0.95 (0.86-1.05) | 0.81 (0.61-1.09) | 0.18 |
|  |  | Adj. for demographic and lifestyle factors | 1.06 (0.91-1.23) | 1.00 (Ref.) | 0.92 (0.822-1.03) | 0.76 (0.56-1.05) | 0.13 |
|  | 55-64 years (n cases) | | 1,276 | 8,418 | 2,616 | 440 |  |
|  |  | Unadjusted | 1.21 (1.12-1.31) | 1.00 (Ref.) | 1.07 (1.02-1.14) | 1.27 (1.12-1.45) | < 0.001 |
|  |  | Adj. for demographics | 1.21 (1.12-1.31) | 1.00 (Ref.) | 1.07 (1.01-1.13) | 1.21 (1.06-1.38) | < 0.001 |
|  |  | Adj. for demographic and lifestyle factors | 1.19 (1.09-1.29) | 1.00 (Ref.) | 1.08 (1.01-1.15) | 1.18 (1.02-1.36) | < 0.001 |
|  | 65-74 years (n cases) | | 1,150 | 8,570 | 2,555 | 605 |  |
|  |  | Unadjusted | 1.06 (0.98-1.15) | 1.00 (Ref.) | 1.00 (0.94-1.06) | 1.31 (1.17-1.47) | < 0.001 |
|  |  | Adj. for demographics | 1.06 (0.97-1.15) | 1.00 (Ref.) | 0.98 (0.92-1.04) | 1.24 (1.11-1.40) | 0.002 |
|  |  | Adj. for demographic and lifestyle factors | 1.05 (0.96-1.15) | 1.00 (Ref.) | 0.97 (0.91-1.04) | 1.26 (1.11-1.43) | 0.002 |
|  | ≥75 years (n cases) | | 246 | 1,569 | 521 | 111 |  |
|  |  | Unadjusted | 1.20 (1.00-1.44) | 1.00 (Ref.) | 1.18 (1.04-1.36) | 1.02 (0.79-1.31) | 0.04 |
|  |  | Adj. for demographics | 1.22 (1.01-1.48) | 1.00 (Ref.) | 1.19 (1.03-1.36) | 0.98 (0.76-1.27) | 0.03 |
|  |  | Adj. for demographic and lifestyle factors | 1.22 (0.99-1.50) | 1.00 (Ref.) | 1.21 (1.04-1.40) | 0.94 (0.71-1.24) | 0.03 |
| Results presented as odds ratio (95% confidence interval) and p-value. Demographic factors include age, race, ethnicity, region, and BMI. Lifestyle factors include smoking status, education, normalized socioeconomic status (NSES), and alcohol use. | | | | | | | |

|  | **Supplementary Table S5: Results from logistic regression analyses for the associations between birthweight and age at events related to menarche and menopause, stratified by self-reported race and ethnicity** | | | | | | |
| --- | --- | --- | --- | --- | --- | --- | --- |
|  |  |  | **Birth weight category**  **OR (95% CI)** | | | |  |
|  |  |  | **< 6 lbs.** | **6 lbs. – 7 lbs. 15 oz.** | **8 lbs. – 9 lbs. 15 oz.** | **≥ 10 lbs.** | **Global P** |
| Were periods regular | | |  |  |  |  |  |
|  | Asian/PI (n cases) | | N = 271 | N = 1,127 | N = 141 | N = <20 |  |
|  |  | Unadjusted | 0.73 (0.54-0.98) | 1.00 (Ref.) | 0.93 (0.61-1.41) | 0.63 (0.23-1.74) | 0.18 |
|  |  | Adj. for demographics | 0.74 (0.53-1.03) | 1.00 (Ref.) | 0.83 (0.51-1.33) | 0.57 (0.18-1.78) | 0.26 |
|  |  | Adj. for demographic and lifestyle factors | 0.79 (0.55-1.14) | 1.00 (Ref.) | 0.78 (0.47-1.29) | 0.65 (0.18-2.38) | 0.47 |
|  | Black (n cases) | | N = 589 | N = 3,407 | N = 736 | N = 137 |  |
|  |  | Unadjusted | 0.72 (0.58-0.90) | 1.00 (Ref.) | 0.85 (0.69-1.05) | 0.93 (0.58-1.49) | 0.02 |
|  |  | Adj. for demographics | 0.71 (0.57-0.88) | 1.00 (Ref.) | 0.91 (0.73-1.13) | 0.93 (0.58-1.49) | 0.02 |
|  |  | Adj. for demographic and lifestyle factors | 0.77 (0.60-1.00) | 1.00 (Ref.) | 0.91 (0.71-1.17) | 0.99 (0.57-1.73) | 0.27 |
|  | White (n cases) | | N = 4,563 | N = 37,499 | N = 11,493 | N = 1,922 |  |
|  |  | Unadjusted | 0.86 (0.80-0.92) | 1.00 (Ref.) | 0.97 (0.92-1.02) | 0.87 (0.78-0.96) | < 0.001 |
|  |  | Adj. for demographics | 0.87 (0.81-0.93) | 1.00 (Ref.) | 0.97 (0.92-1.02) | 0.85 (0.77-0.95) | < 0.001 |
|  |  | Adj. for demographic and lifestyle factors | 0.85 (0.79-0.92) | 1.00 (Ref.) | 0.97 (0.92-1.03) | 0.86 (0.77-0.96) | < 0.001 |
|  | Other (n cases) | | N = 84 | N = 532 | N = 163 | N = 27 |  |
|  |  | Unadjusted | 0.73 (0.46-1.16) | 1.00 (Ref.) | 1.25 (0.83-1.90) | 0.64 (0.31-1.32) | 0.17 |
|  |  | Adj. for demographics | 0.68 (0.42-1.11) | 1.00 (Ref.) | 1.26 (0.80-1.97) | 0.84 (0.36-1.96) | 0.24 |
|  |  | Adj. for demographic and lifestyle factors | 0.61 (0.36-1.04) | 1.00 (Ref.) | 1.18 (0.73-1.92) | 0.95 (0.37-2.45) | 0.23 |
|  | Hispanic/Latinx (n cases) | | N = 288 | N = 1,713 | N = 428 | N = 66 |  |
|  |  | Unadjusted | 0.87 (0.65-1.15) | 1.00 (Ref.) | 1.19 (0.91-1.55) | 0.82 (0.47-1.41) | 0.31 |
|  |  | Adj. for demographics | 0.87 (0.59-1.27) | 1.00 (Ref.) | 1.27 (0.90-1.81) | 0.90 (0.44-1.83) | 0.42 |
|  |  | Adj. for demographic and lifestyle factors | 0.77 (0.52-1.15) | 1.00 (Ref.) | 1.29 (0.89-1.88) | 0.70 (0.34-1.45) | 0.17 |
|  | Not Hispanic/Latinx (n cases) | | N = 5,287 | N = 41,320 | N = 12,246 | N = 2,057 |  |
|  |  | Unadjusted | 0.85 (0.79-0.90) | 1.00 (Ref.) | 0.95 (0.91-1.00) | 0.85 (0.77-0.94) | < 0.001 |
|  |  | Adj. for demographics | 0.84 (0.79-0.90) | 1.00 (Ref.) | 0.96 (0.92-1.01) | 0.85 (0.77-0.94) | < 0.001 |
|  |  | Adj. for demographic and lifestyle factors | 0.84 (0.78-0.90) | 1.00 (Ref.) | 0.97 (0.92-1.02) | 0.87 (0.78-0.97) | < 0.001 |
|  | | | | | | | |
| Ever had premenopausal hot flashes | | |  |  |  |  |  |
|  | Asian (n cases) | | N = 281 | N = 1,130 | N = 145 | N = <20 |  |
|  |  | Unadjusted | 0.81 (0.60-1.10) | 1.00 (Ref.) | 1.08 (0.70-1.68) | 0.49 (0.19-1.26) | 0.25 |
|  |  | Adj. for demographic | 0.78 (0.55-1.10) | 1.00 (Ref.) | 1.21 (0.73-2.01) | 0.50 (0.17-1.44) | 0.22 |
|  |  | Adj. for demographic and lifestyle factors | 0.78 (0.54-1.12) | 1.00 (Ref.) | 1.21 (0.70-2.07) | 0.35 (0.11-1.07) | 0.12 |
|  | Black (n cases) | | N = 577 | N = 3,150 | N = 688 | N = 127 |  |
|  |  | Unadjusted | 1.07 (0.87-1.31) | 1.00 (Ref.) | 0.96 (0.80-1.15) | 0.92 (0.63-1.36) | 0.80 |
|  |  | Adj. for demographics | 1.05 (0.86-1.29) | 1.00 (Ref.) | 0.96 (0.79-1.15) | 0.90 (0.61-1.33) | 0.84 |
|  |  | Adj. for demographic and lifestyle factors | 0.99 (0.78-1.24) | 1.00 (Ref.) | 0.92 (0.75-1.12) | 0.87 (0.56-1.35) | 0.79 |
|  | White (n cases) | | N = 4,750 | N = 38,227 | N = 11,843 | N = 2,023 |  |
|  |  | Unadjusted | 0.95 (0.88-1.02) | 1.00 (Ref.) | 1.03 (0.98-1.09) | 1.02 (0.91-1.14) | 0.21 |
|  |  | Adj. for demographics | 0.95 (0.88-1.02) | 1.00 (Ref.) | 1.04 (0.98-1.09) | 1.00 (0.90-1.12) | 0.20 |
|  |  | Adj. for demographic and lifestyle factors | 0.97 (0.89-1.05) | 1.00 (Ref.) | 1.03 (0.97-1.09) | 1.05 (0.93-1.19) | 0.44 |
|  | Other (n cases) | | N = 87 | N = 548 | N = 161 | N = 33 |  |
|  |  | Unadjusted | 0.74 (0.46-1.18) | 1.00 (Ref.) | 0.97 (0.65-1.45) | 1.25 (0.51-3.06) | 0.57 |
|  |  | Adj. for demographics | 0.76 (0.46-1.27) | 1.00 (Ref.) | 1.00 (0.65-1.55) | 1.05 (0.42-2.62) | 0.76 |
|  |  | Adj. for demographic and lifestyle factors | 0.77 (0.44-1.37) | 1.00 (Ref.) | 1.01 (0.63-1.61) | 1.54 (0.52-4.59) | 0.68 |
|  | Hispanic/Latinx (n cases) | | N = 295 | N = 1,693 | N = 419 | N = 70 |  |
|  |  | Unadjusted | 1.03 (0.78-1.37) | 1.00 (Ref.) | 1.11 (0.86-1.42) | 1.19 (0.66-2.13) | 0.83 |
|  |  | Adj. for demographics | 1.08 (0.73-1.57) | 1.00 (Ref.) | 0.98 (0.72-1.34) | 0.85 (0.44-1.65) | 0.94 |
|  |  | Adj. for demographic and lifestyle factors | 0.98 (0.65-1.46) | 1.00 (Ref.) | 0.99 (0.71-1.37) | 0.75 (0.37-1.52) | 0.88 |
|  | Not Hispanic/Latinx (n cases) | | N = 5,471 | N = 41,821 | N = 12,564 | N = 2,154 |  |
|  |  | Unadjusted | 0.94 (0.88-1.01) | 1.00 (Ref.) | 1.03 (0.98-1.09) | 1.02 (0.92-1.13) | 0.12 |
|  |  | Adj. for demographics | 0.95 (0.88-1.01) | 1.00 (Ref.) | 1.03 (0.98-1.08) | 0.99 (0.89-1.10) | 0.20 |
|  |  | Adj. for demographic and lifestyle factors | 0.96 (0.89-1.03) | 1.00 (Ref.) | 1.02 (0.97-1.08) | 1.04 (0.92-1.08) | 0.46 |
|  |  |  |  |  |  |  |  |
| Ever had postmenopausal hot flashes | | |  |  |  |  |  |
|  | Asian (n cases) | | N = 92 | N = 344 | N = 36 | N = <20 |  |
|  |  | Unadjusted | 1.00 (0.75-1.32) | 1.00 (Ref.) | 0.84 (0.56-1.27) | 2.33 (0.84-6.74) | 0.33 |
|  |  | Adj. for demographic | 1.04 (0.76-1.43) | 1.00 (Ref.) | 0.67 (0.41-1.09) | 2.03 (0.64-6.49) | 0.23 |
|  |  | Adj. for demographic and lifestyle factors | 1.08 (0.77-1.52) | 1.00 (Ref.) | 0.55 (0.32-0.95) | 1.81 (0.52-6.26) | 0.10 |
|  | Black (n cases) | | N = 270 | N = 1,561 | N = 344 | N = 62 |  |
|  |  | Unadjusted | 0.88 (0.72-1.08) | 1.00 (Ref.) | 1.04 (0.87-1.26) | 1.10 (0.73-1.68) | 0.53 |
|  |  | Adj. for demographics | 0.90 (0.74-1.10) | 1.00 (Ref.) | 1.07 (0.88-1.30) | 1.13 (0.74-1.74) | 0.56 |
|  |  | Adj. for demographic and lifestyle factors | 0.95 (0.75-1.20) | 1.00 (Ref.) | 1.09 (0.88-1.36) | 1.27 (0.77-2.11) | 0.62 |
|  | White (n cases) | | N = 1,883 | N = 15,092 | N = 4,648 | N = 781 |  |
|  |  | Unadjusted | 1.00 (0.94-1.07) | 1.00 (Ref.) | 0.98 (0.94-1.03) | 0.92 (0.83-1.01) | 0.31 |
|  |  | Adj. for demographics | 1.01 (0.94-1.08) | 1.00 (Ref.) | 0.98 (0.94-1.03) | 0.98 (0.88-1.08) | 0.80 |
|  |  | Adj. for demographic and lifestyle factors | 1.00 (0.93-1.07) | 1.00 (Ref.) | 0.99 (0.94-1.04) | 0.96 (0.86-1.06) | 0.84 |
|  | Other (n cases) | | N = 42 | N = 237 | N = 62 | N = <20 |  |
|  |  | Unadjusted | 1.24 (0.75-2.04) | 1.00 (Ref.) | 0.90 (0.60-1.33) | 1.42 (0.60-3.34) | 0.62 |
|  |  | Adj. for demographics | 1.34 (0.78-2.30) | 1.00 (Ref.) | 0.82 (0.54-1.27) | 1.49 (0.57-3.86) | 0.39 |
|  |  | Adj. for demographic and lifestyle factors | 1.25 (0.68-2.29) | 1.00 (Ref.) | 0.80 (0.50-1.28) | 1.07 (0.39-2.92) | 0.64 |
|  | Hispanic/Spanish/Latinx (n cases) | | N = 122 | N = 717 | N = 152 | N = 28 |  |
|  |  | Unadjusted | 1.00 (0.76-1.31) | 1.00 (Ref.) | 0.84 (0.66-1.07) | 0.86 (0.51-1.46) | 0.53 |
|  |  | Adj. for demographics | 0.75 (0.52-1.09) | 1.00 (Ref.) | 0.84 (0.62-1.15) | 0.98 (0.49-1.96) | 0.38 |
|  |  | Adj. for demographic and lifestyle factors | 0.78 (0.53-1.15) | 1.00 (Ref.) | 0.89 (0.64-1.23) | 0.90 (0.42-1.92) | 0.60 |
|  | Not Hispanic/Spanish/Latinx (n cases) | | N = 2,208 | N = 16,690 | N = 4,978 | N = 846 |  |
|  |  | Unadjusted | 1.00 (0.94-1.07) | 1.00 (Ref.) | 0.99 (0.94-1.03) | 0.94 (0.85-1.03) | 0.58 |
|  |  | Adj. for demographics | 1.01 (0.95-1.08) | 1.00 (Ref.) | 0.98 (0.94-1.03) | 1.00 (0.90-1.10) | 0.87 |
|  |  | Adj. for demographic and lifestyle factors | 1.01 (0.94-1.08) | 1.00 (Ref.) | 0.99 (0.94-1.04) | 0.98 (0.88-1.09) | 0.92 |
|  |  |  |  |  |  |  |  |
| Ever had an oophorectomy | | |  |  |  |  |  |
|  | Asian (n cases) | | N = 95 | N = 352 | N = 47 | N = <20 |  |
|  |  | Unadjusted | 1.05 (0.81-1.37) | 1.00 (Ref.) | 1.08 (0.76-1.55) | 1.39 (0.56-3.48) | 0.86 |
|  |  | Adj. for demographic | 1.27 (0.95-1.71) | 1.00 (Ref.) | 1.21 (0.81-1.82) | 1.71 (0.65-4.48) | 0.26 |
|  |  | Adj. for demographic and lifestyle factors | 1.21 (0.89-1.66) | 1.00 (Ref.) | 1.25 (0.82-1.93) | 2.35 (0.83-6.71) | 0.22 |
|  | Black (n cases) | | N = 257 | N = 1,470 | N = 321 | N = 57 |  |
|  |  | Unadjusted | 0.94 (0.80-1.12) | 1.00 (Ref.) | 0.97 (0.83-1.13) | 0.90 (0.65-1.26) | 0.84 |
|  |  | Adj. for demographics | 0.96 (0.81-1.14) | 1.00 (Ref.) | 0.97 (0.83-1.13) | 0.88 (0.63-1.23) | 0.84 |
|  |  | Adj. for demographic and lifestyle factors | 0.97 (0.80-1.17) | 1.00 (Ref.) | 0.96 (0.81-1.15) | 1.06 (0.72-1.55) | 0.94 |
|  | White (n cases) | | N = 1,756 | N = 12,582 | N = 4,015 | N = 762 |  |
|  |  | Unadjusted | 1.17 (1.10-1.24) | 1.00 (Ref.) | 1.05 (1.01-1.09) | 1.23 (1.12-1.34) | < 0.001 |
|  |  | Adj. for demographics | 1.16 (1.10-1.24) | 1.00 (Ref.) | 1.03 (0.98-1.07) | 1.16 (1.06-1.27) | < 0.001 |
|  |  | Adj. for demographic and lifestyle factors | 1.16 (1.08-1.23) | 1.00 (Ref.) | 1.03 (0.98-1.08) | 1.14 (1.04-1.25) | < 0.001 |
|  | Other (n cases) | | N = 44 | N = 219 | N = 62 | N = <20 |  |
|  |  | Unadjusted | 1.34 (0.89-2.03) | 1.00 (Ref.) | 0.94 (0.67-1.32) | 0.44 (0.19-1.01) | 0.10 |
|  |  | Adj. for demographics | 1.29 (0.82-2.03) | 1.00 (Ref.) | 0.98 (0.68-1.42) | 0.31 (0.12-0.82) | 0.06 |
|  |  | Adj. for demographic and lifestyle factors | 1.29 (0.78-2.13) | 1.00 (Ref.) | 1.00 (0.67-1.49) | 0.36 (0.13-0.98) | 0.15 |
|  | Hispanic/Latinx (n cases) | | N = 98 | N = 558 | N = 125 | N = 26 |  |
|  |  | Unadjusted | 1.00 (0.78-1.29) | 1.00 (Ref.) | 0.89 (0.71-1.11) | 1.24 (0.77-1.99) | 0.56 |
|  |  | Adj. for demographics | 1.18 (0.85-1.64) | 1.00 (Ref.) | 0.78 (0.58-1.04) | 1.28 (0.70-2.34) | 0.16 |
|  |  | Adj. for demographic and lifestyle factors | 1.19 (0.84-1.69) | 1.00 (Ref.) | 0.73 (0.53-1.01) | 1.47 (0.77-2.80) | 0.07 |
|  | Not Hispanic/Latinx (n cases) | | N = 2,076 | N = 14,209 | N = 4,373 | N = 815 |  |
|  |  | Unadjusted | 1.16 (1.10-1.23) | 1.00 (Ref.) | 1.04 (1.00-1.08) | 1.18 (1.08-1.28) | < 0.001 |
|  |  | Adj. for demographics | 1.14 (1.08-1.21) | 1.00 (Ref.) | 1.03 (0.99-1.07) | 1.12 (1.03-1.23) | < 0.001 |
|  |  | Adj. for demographic and lifestyle factors | 1.14 (1.07-1.21) | 1.00 (Ref.) | 1.03 (0.99-1.08) | 1.13 (1.03-1.23) | < 0.001 |
|  |  |  |  |  |  |  |  |
| Ever had a hysterectomy | | |  |  |  |  |  |
|  | Asian (n cases) | | N = 119 | N = 442 | N = 63 | N = <20 |  |
|  |  | Unadjusted | 1.06 (0.83-1.36) | 1.00 (Ref.) | 1.19 (0.86-1.66) | 1.86 (0.78-4.41) | 0.39 |
|  |  | Adj. for demographic | 1.29 (0.98-1.71) | 1.00 (Ref.) | 1.27 (0.87-1.85) | 2.01 (0.78-5.16) | 0.12 |
|  |  | Adj. for demographic and lifestyle factors | 1.21 (0.90-1.63) | 1.00 (Ref.) | 1.23 (0.81-1.85) | 2.01 (0.70-5.78) | 0.31 |
|  | Black (n cases) | | N = 388 | N = 2,165 | N = 465 | N = 89 |  |
|  |  | Unadjusted | 0.98 (0.84-1.15) | 1.00 (Ref.) | 0.95 (0.82-1.10) | 1.01 (0.73-1.38) | 0.90 |
|  |  | Adj. for demographics | 1.00 (0.85-1.18) | 1.00 (Ref.) | 0.95 (0.82-1.10) | 0.99 (0.72-1.37) | 0.92 |
|  |  | Adj. for demographic and lifestyle factors | 1.05 (0.87-1.27) | 1.00 (Ref.) | 0.91 (0.77-1.08) | 1.25 (0.85-1.83) | 0.40 |
|  | White (n cases) | | N = 2,462 | N = 18,164 | N = 5,783 | N = 1,098 |  |
|  |  | Unadjusted | 1.14 (1.08-1.21) | 1.00 (Ref.) | 1.06 (1.02-1.10) | 1.27 (1.17-1.38) | < 0.001 |
|  |  | Adj. for demographics | 1.13 (1.07-1.20) | 1.00 (Ref.) | 1.03 (0.99-1.07) | 1.18 (1.09-1.29) | < 0.001 |
|  |  | Adj. for demographic and lifestyle factors | 1.11 (1.04-1.18) | 1.00 (Ref.) | 1.03 (0.99-1.07) | 1.14 (1.05-1.25) | < 0.001 |
|  | Other (n cases) | | N = 69 | N = 325 | N = 90 | N = <20 |  |
|  |  | Unadjusted | 1.61 (1.08-2.41) | 1.00 (Ref.) | 0.89 (0.65-1.22) | 0.60 (0.31-1.18) | 0.03 |
|  |  | Adj. for demographics | 1.66 (1.08-2.56) | 1.00 (Ref.) | 0.90 (0.64-1.27) | 0.47 (0.22-1.00) | 0.02 |
|  |  | Adj. for demographic and lifestyle factors | 1.78 (1.09-2.89) | 1.00 (Ref.) | 0.87 (0.60-1.26) | 0.49 (0.22-1.10) | 0.02 |
|  | Hispanic/Latinx (n cases) | | N = 144 | N = 921 | N = 217 | N = 40 |  |
|  |  | Unadjusted | 0.84 (0.67-1.05) | 1.00 (Ref.) | 0.95 (0.78-1.16) | 1.16 (0.75-1.79) | 0.39 |
|  |  | Adj. for demographics | 0.88 (0.64-1.19) | 1.00 (Ref.) | 0.90 (0.70-1.16) | 0.95 (0.54-1.67) | 0.75 |
|  |  | Adj. for demographic and lifestyle factors | 0.80 (0.58-1.12) | 1.00 (Ref.) | 0.83 (0.64-1.10) | 1.03 (0.55-1.92) | 0.40 |
|  | Not Hispanic/Latinx (n cases) | | N = 2,936 | N = 20,427 | N = 6,264 | N = 1,187 |  |
|  |  | Unadjusted | 1.16 (1.10-1.22) | 1.00 (Ref.) | 1.04 (1.01-1.08) | 1.24 (1.15-1.35) | < 0.001 |
|  |  | Adj. for demographics | 1.14 (1.08-1.20) | 1.00 (Ref.) | 1.03 (0.99-1.07) | 1.17 (1.08-1.27) | < 0.001 |
|  |  | Adj. for demographic and lifestyle factors | 1.12 (1.06-1.19) | 1.00 (Ref.) | 1.03 (0.99-1.07) | 1.15 (1.05-1.26) | < 0.001 |
| Results presented as odds ratio (95% confidence interval) and p-value. Demographic factors include age, race, ethnicity, region, and BMI. Lifestyle factors include smoking status, education, normalized socioeconomic status (NSES), and alcohol use. | | | | | | | |

| **Supplementary Table S6: Results from linear regression analyses for the associations between preterm birth status and age at events related to menarche and menopause, stratified by birth weight** | | | | | | |
| --- | --- | --- | --- | --- | --- | --- |
|  |  | **Birth weight category**  **β (SE) P** | | | |  |
|  |  | **< 6 lbs.** | **6 lbs. – 7 lbs. 15 oz.** | **8 lbs. – 9 lbs. 15 oz.** | **≥ 10 lbs.** | **P(intxn)** |
| N (preterm/term) | | 1,687/6,356 | 277/51,728 | 29/15,414 | <20/2,638 |  |
| Age at first regular period | |  |  |  |  |  |
|  | Unadjusted | -0.012 (0.06)  0.84 | 0.033 (0.12)  0.78 | 0.340 (0.40)  0.40 | --- | 0.23 |
|  | Adj. for demographics | 0.013 (0.06)  0.83 | 0.015 (0.12)  0.90 | 0.444 (0.41)  0.27 | --- | 0.36 |
|  | Adj. for demographic and lifestyle factors | -0.003 (0.06)  0.96 | -0.001 (0.13)  0.99 | 0.205 (0.45)  0.65 | --- | 0.57 |
| Age at last regular period | |  |  |  |  |  |
|  | Unadjusted | -0.104 (0.20)  0.61 | -0.720 (0.42)  0.08 | 0.309 (1.27)  0.81 | --- | 0.87 |
|  | Adj. for demographics | 0.032 (0.20)  0.88 | -0.575 (0.41)  0.16 | 0.480 (1.28)  0.71 | --- | 0.86 |
|  | Adj. for demographic and lifestyle factors | 0.059 (0.22)  0.78 | -0.658 (0.43)  0.13 | 0.032 (1.37)  0.98 | --- | 0.65 |
| Age at last vaginal bleeding | |  |  |  |  |  |
|  | Unadjusted | -0.152 (0.22)  0.48 | -0.406 (0.46)  0.38 | 0.404 (1.41)  0.77 | --- | 0.30 |
|  | Adj. for demographics | -0.021 (0.22)  0.92 | -0.259 (0.46)  0.57 | 0.557 (1.42)  0.69 | --- | 0.32 |
|  | Adj. for demographic and lifestyle factors | 0.012 (0.23)  0.96 | -0.219 (0.48)  0.65 | 0.138 (1.52)  0.93 | --- | 0.61 |
| Age at menarche | |  |  |  |  |  |
|  | Unadjusted | -0.053 (0.04)  0.21 | 0.070 (0.09)  0.43 | 0.095 (0.27)  0.73 | --- | 0.15 |
|  | Adj. for demographics | 0.008 (0.04)  0.86 | 0.080 (0.09)  0.36 | 0.165 (0.27)  0.54 | --- | 0.31 |
|  | Adj. for demographic and lifestyle factors | -0.005 (0.05)  0.91 | 0.077 (0.09)  0.41 | 0.056 (0.29)  0.85 | --- | 0.35 |
| Age at natural menopause | |  |  |  |  |  |
|  | Unadjusted | -0.279 (0.19)  0.15 | -0.359 (0.39)  0.35 | -0.352 (1.30)  0.79 | --- | 0.43 |
|  | Adj. for demographic | -0.183 (0.20)  0.35 | -0.165 (0.39)  0.67 | 0.045 (1.32)  0.97 | --- | 0.29 |
|  | Adj. for demographic and lifestyle factors | -0.225 (0.21)  0.28 | -0.131 (0.40)  0.75 | -0.782 (1.42)  0.58 | --- | 0.52 |
| Age at nat. menopause (conservative) | |  |  |  |  |  |
|  | Unadjusted | -0.267 (0.21)  0.20 | -0.656 (0.42)  0.12 | -0.399 (1.30)  0.76 | --- | 0.86 |
|  | Adj. for demographics | -0.170 (0.21)  0.43 | -0.483 (0.42)  0.25 | 0.039 (1.32)  0.98 | --- | 0.68 |
|  | Adj. for demographic and lifestyle factors | -0.196 (0.22)  0.38 | -0.421 (0.44)  0.34 | -0.791 (1.42)  0.58 | --- | 0.98 |
| Reproductive window | |  |  |  |  |  |
|  | Unadjusted | -0.217 (0.21)  0.31 | -0.736 (0.43)  0.09 | -0.658 (1.33)  0.62 | --- | 0.87 |
|  | Adj. for demographics | -0.175 (0.22)  0.42 | -0.587 (0.43)  0.17 | -0.361 (1.35)  0.79 | --- | 0.94 |
|  | Adj. for demographic and lifestyle factors | -0.199 (0.23)  0.39 | -0.517 (0.45)  0.25 | -1.249 (1.45)  0.39 | --- | 0.77 |
| Results presented as beta (standard error) and p-value. Demographic factors include age, race, ethnicity, region, and BMI. Lifestyle factors include smoking status, education, normalized socioeconomic status (NSES), and alcohol use. For the age at natural menopause analyses, participants were removed if they reported: having a bilateral oophorectomy prior to menopause; having an oophorectomy prior to menopause but did not know if it was unilateral or bilateral; or if they had an unknown oophorectomy status. For the conservative age at natural menopause, participants were also removed if they reported having a hysterectomy prior to menopause or had an unknown hysterectomy status. | | | | | | |

| **Supplementary Table S7: Results from linear regression analyses for the associations between preterm birth status and events related to menarche and menopause, stratified by age at enrollment** | | | | | |
| --- | --- | --- | --- | --- | --- |
|  | | **45-54 years β (SE)**  **P** | **55-64 years β (SE)**  **P** | **65-74 years β (SE)**  **P** | **≥ 75 years β (SE)**  **P** |
| N (preterm/term) | | 357/11,359 | 916/34,348 | 660/32,863 | 109/6,313 |
| Age at first regular period | |  |  |  |  |
|  | Unadjusted | 0.022 (0.12)  0.85 | 0.014 (0.07)  0.85 | -0.059 (0.08)  0.44 | 0.108 (0.18)  0.56 |
|  | Adj for demographics | -0.024 (0.12)  0.84 | 0.030 (0.07)  0.67 | -0.043 (0.08)  0.57 | 0.137 (0.18)  0.46 |
|  | Adj for demographics and lifestyle factors | -0.103 (0.13)  0.42 | 0.029 (0.07)  0.70 | -0.046 (0.08) 0.57 | 0.213 (0.20)  0.28 |
|  |  |  |  |  |  |
| Age at last regular period | |  |  |  |  |
|  | Unadjusted | -0.352 (0.34)  0.30 | -0.611 (0.24)  0.01 | -0.412 (0.27)  0.13 | -0.609 (0.65)  0.35 |
|  | Adj. for demographics | -0.203 (0.34)  0.55 | -0.528 (0.24)  0.03 | -0.374 (0.27)  0.17 | -0.375 (0.65)  0.57 |
|  | Adj. for demographics and lifestyle factors | -0.401 (0.37)  0.28 | -0.418 (0.25)  0.10 | -0.331 (0.29)  0.25 | -0.156 (0.69)  0.82 |
|  |  |  |  |  |  |
| Age at last vaginal bleeding | |  |  |  |  |
|  | Unadjusted | -0.703 (0.35)  0.047 | -0.807 (0.26)  0.002 | -0.277 (0.31)  0.37 | -0.535 (0.75)  0.48 |
|  | Adj. for demographics | -0.477 (0.36)  0.18 | -0.709 (0.26)  0.006 | -0.262 (0.31)  0.40 | -0.271 (0.76)  0.72 |
|  | Adj. for demographics and lifestyle factors | -0.571 (0.38)  0.14 | -0.545 (0.27)  0.046 | -0.233 (0.32)  0.47 | 0.124 (0.80)  0.88 |
|  |  |  |  |  |  |
| Age at menarche | |  |  |  |  |
|  | Unadjusted | -0.107 (0.08)  0.19 | -0.013 (0.05)  0.79 | -0.103 (0.06)  0.07 | 0.111 (0.14)  0.44 |
|  | Adj. for demographics | -0.079 (0.08)  0.34 | 0.011 (0.05)  0.82 | -0.091 (0.06)  0.11 | 0.143 (0.14)  0.32 |
|  | Adj. for demographics and lifestyle factors | -0.128 (0.09)  0.15 | -0.001 (0.05)  0.98 | -0.089 (0.06)  0.14 | 0.179 (0.15)  0.24 |
|  |  |  |  |  |  |
| Reproductive window | |  |  |  |  |
|  | Unadjusted | -0.258 (0.34)  0.45 | -0.905 (0.24)  < 0.001 | -0.346 (0.30)  0.25 | -0.608 (0.72)  0.40 |
|  | Adj. for demographics | -0.054 (0.34)  0.87 | -0.822 (0.24)  < 0.001 | -0.339 (0.30)  0.26 | -0.384 (0.74)  0.60 |
|  | Adj. for demographics and lifestyle factors | 0.151 (0.37)  0.69 | -0.715 (0.25)  0.004 | -0.397 (0.32)  0.21 | -0.557 (0.76)  0.47 |
|  |  |  |  |  |  |
| Age at natural menopause | |  |  |  |  |
|  | Unadjusted | -0.358 (0.29)  0.21 | -0.944 (0.21)  < 0.001 | -0.328 (0.28)  0.25 | -0.251 (0.68)  0.71 |
|  | Adj. for demographics | -0.192 (0.29)  0.51 | -0.821 (0.21)  < 0.001 | -0.307 (0.28)  0.28 | -0.024 (0.69)  0.97 |
|  | Adj. for demographics and lifestyle factors | -0.044 (0.31)  0.89 | -0.766 (0.23)  < 0.001 | -0.372 (0.30)  0.21 | -0.093 (0.72)  0.90 |
|  |  |  |  |  |  |
| Age at natural menopause (conservative) | |  |  |  |  |
|  | Unadjusted | -0.337 (0.33)  0.31 | -0.902 (0.23)  < 0.001 | -0.428 (0.30)  0.15 | -0.501 (0.71)  0.48 |
|  | Adj. for demographics | -0.147 (0.33)  0.65 | -0.785 (0.23)  < 0.001 | -0.402 (0.30)  0.18 | -0.249 (0.72)  0.73 |
|  | Adj. for demographics and lifestyle factors | -0.010 (0.36)  0.98 | -0.694 (0.24)  0.004 | -0.430 (0.31)  0.16 | -0.349 (0.75)  0.64 |
| Results presented as beta (standard error). Demographic factors include age, race, ethnicity, region, and BMI. Lifestyle factors include smoking status, education, normalized socioeconomic status (NSES), and alcohol use. For the age at natural menopause analyses, participants were removed if they reported: having a bilateral oophorectomy prior to menopause; having an oophorectomy prior to menopause but did not know if it was unilateral or bilateral; or if they had an unknown oophorectomy status. For the conservative age at natural menopause, participants were also removed if they reported having a hysterectomy prior to menopause or had an unknown hysterectomy status. | | | | | |

| **Supplementary Table S8: Results from linear regression analyses for the associations between preterm birth status and events related to menarche and menopause, stratified by self-reported race and ethnicity** | | | | | | | |
| --- | --- | --- | --- | --- | --- | --- | --- |
|  | | **Race** | | | | **Ethnicity** | |
|  |  | **Asian/PI β (SE)**  **P** | **Black β (SE)**  **P** | **White β (SE)**  **P** | **Other β (SE)**  **P** | **Hispanic/Latinx β (SE)**  **P** | **Not Hispanic/Latinx β (SE)**  **P** |
| N (preterm/term) | | 44/2,327 | 162/6,621 | 1,770/72,954 | 26/1,135 | 83/3,588 | 1,941/80,472 |
| Age at first regular period | |  |  |  |  |  |  |
|  | Unadjusted | 0.318 (0.32)  0.32 | 0.313 (0.17)  0.07 | -0.064 (0.05)  0.18 | 0.745 (0.43)  0.08 | -0.051 (0.23)  0.83 | -0.013 (0.05)  0.77 |
|  | Adj for demographics | 0.395 (0.33)  0.24 | 0.318 (0.17)  0.06 | -0.040 (0.05) 0.41 | 0.651 (0.48)  0.18 | -0.111 (0.29)  0.70 | 0.004 (0.05)  0.93 |
|  | Adj for demographics and lifestyle factors | 0.526 (0.36)  0.14 | 0.283 (0.20)  0.15 | -0.048 (0.05)  0.35 | 0.501 (0.49)  0.30 | -0.104 (0.30)  0.73 | -0.007 (0.05)  0.89 |
|  |  |  |  |  |  |  |  |
| Age at last regular period | |  |  |  |  |  |  |
|  | Unadjusted | 0.202 (0.99)  0.84 | -0.121 (0.62)  0.85 | -0.717 (0.17)  < 0.001 | 0.512 (1.44)  0.72 | -0.221 (0.86)  0.80 | -0.653 (0.16)  < 0.001 |
|  | Adj. for demographics | 0.479 (1.06)  0.65 | 0.271 (0.64)  0.67 | -0.539 (0.16)  0.001 | 0.620 (1.58)  0.70 | -0.002 (1.02)  1.00 | -0.461 (0.16)  0.004 |
|  | Adj. for demographics and lifestyle factors | 0.815 (1.13)  0.47 | 0.434 (0.72)  0.55 | -0.501 (0.17)  0.004 | 0.820 (1.62)  0.61 | 0.852 (1.08)  0.43 | -0.437 (0.17)  0.009 |
|  |  |  |  |  |  |  |  |
| Age at last bleeding | |  |  |  |  |  |  |
|  | Unadjusted | 0.325 (1.02)  0.75 | -0.563 (0.64)  0.38 | -0.820 (0.18)  < 0.001 | 1.648 (1.53)  0.28 | -1.184 (0.89)  0.19 | -0.757 (0.18)  < 0.001 |
|  | Adj. for demographics | 0.336 (1.08)  0.76 | -0.206 (0.65)  0.75 | -0.619 (0.18)  < 0.001 | 2.342 (1.69)  0.17 | -0.624 (1.09)  0.57 | -0.535 (0.17)  0.002 |
|  | Adj. for demographics and lifestyle factors | 0.746 (1.17)  0.52 | 0.115 (0.74)  0.88 | -0.549 (0.19)  0.004 | 2.784 (1.76)  0.11 | 0.026 (1.17)  0.98 | -0.454 (0.18)  0.01 |
|  |  |  |  |  |  |  |  |
| Age at menarche | |  |  |  |  |  |  |
|  | Unadjusted | -0.066 (0.25)  0.79 | 0.170 (0.13) 0.19 | -0.097 (0.03)  0.005 | 0.278 (0.32)  0.39 | -0.356 (0.18)  0.04 | -0.062 (0.03)  0.07 |
|  | Adj. for demographics | 0.096 (0.26)  0.71 | 0.179 (0.13)  0.17 | -0.055 (0.03) 0.12 | 0.231 (0.36)  0.52 | -0.284 (0.22)  0.19 | -0.024 (0.03)  0.47 |
|  | Adj. for demographics and lifestyle factors | 0.206 (0.28)  0.46 | 0.152 (0.15)  0.30 | -0.063 (0.04)  0.09 | 0.091 (0.37)  0.81 | -0.267 (0.23)  0.24 | -0.036 (0.04)  0.31 |
| Age at natural menopause | |  |  |  |  |  |  |
|  | Unadjusted | 0.135 (1.00)  0.89 | -0.920 (0.64)  0.15 | -0.680 (0.16)  < 0.001 | 1.960 (1.45)  0.18 | -0.641 (0.78)  0.41 | -0.678 (0.15)  < 0.001 |
|  | Adj. for demographics | 0.596 (1.04)  0.57 | -0.867 (0.65)  0.18 | -0.558 (0.16)  < 0.001 | 3.066 (1.70)  0.07 | -0.091 (0.96)  0.92 | -0.543 (0.15)  < 0.001 |
|  | Adj. for demographics and lifestyle factors | 0.448 (1.12)  0.69 | -0.632 (0.73)  0.39 | -0.560 (0.16)  < 0.001 | 3.668 (1.87)  0.050 | 0.368 (1.00)  0.71 | -0.531 (0.16)  < 0.001 |
|  |  |  |  |  |  |  |  |
| Age at natural menopause (conservative) | |  |  |  |  |  |  |
|  | Unadjusted | 0.016 (1.00)  0.99 | -1.054 (0.74)  0.15 | -0.664 (0.17)  < 0.001 | 2.115 (1.67)  0.21 | -1.137 (0.92)  0.22 | -0.667 (0.16)  < 0.001 |
|  | Adj. for demographics | 0.434 (1.03)  0.67 | -0.900 (0.75)  0.23 | -0.560 (0.17)  < 0.001 | 3.037 (1.96)  0.12 | -0.467 (1.10)  0.67 | -0.543 (0.16)  < 0.001 |
|  | Adj. for demographics and lifestyle factors | 0.271 (1.11)  0.81 | -0.466 (0.85)  0.58 | -0.546 (0.18)  0.002 | 4.093 (2.21)  0.06 | 0.209 (1.14)  0.86 | -0.507 (0.17)  0.003 |
|  |  |  |  |  |  |  |  |
| Reproductive window | |  |  |  |  |  |  |
|  | Unadjusted | 0.234 (1.04)  0.82 | -1.189 (0.75)  0.11 | -0.602 (0.17)  < 0.001 | 1.944 (1.69)  0.25 | -0.750 (0.95)  0.43 | -0.627 (0.17)  < 0.001 |
|  | Adj. for demographics | 0.446 (1.08)  0.68 | -1.083 (0.76)  0.16 | -0.540 (0.17)  0.002 | 3.348 (1.98)  0.09 | -0.083 (1.14)  0.94 | -0.541 (0.17)  0.001 |
|  | Adj. for demographics and lifestyle factors | 0.244 (1.16)  0.83 | -0.647 (0.87)  0.46 | -0.530 (0.18)  0.003 | 4.838 (2.21)  0.03 | 0.508 (1.19)  0.67 | -0.503 (0.18)  0.004 |
| Results presented as beta (standard error). Demographic factors include age, race, ethnicity, region, and BMI. Lifestyle factors include smoking status, education, normalized socioeconomic status (NSES), and alcohol use. For the age at natural menopause analyses, participants were removed if they reported: having a bilateral oophorectomy prior to menopause; having an oophorectomy prior to menopause but did not know if it was unilateral or bilateral; or if they had an unknown oophorectomy status. For the conservative age at natural menopause, participants were also removed if they reported having a hysterectomy prior to menopause or had an unknown hysterectomy status. | | | | | | | |

| **Supplementary Table S9: Results from logistic regression analyses for the associations between preterm birth status and events related to menarche and menopause, stratified by birthweight** | | | | | | |
| --- | --- | --- | --- | --- | --- | --- |
|  |  | **Birth weight category**  **OR (95% CI) P** | | | |  |
|  |  | **< 6 lbs.** | **6 lbs. – 7 lbs. 15 oz.** | **8 lbs. – 9 lbs. 15 oz.** | **≥ 10 lbs.** | **P (intxn)** |
| N (preterm/term) | | 1,687/6,356 | 277/51,728 | 29/15,414 | <20/2,638 |  |
| Were periods regular | |  |  |  |  |  |
|  | Unadjusted | 0.89 (0.78-1.02)  0.08 | 0.83 (0.61-1.11)  0.20 | 0.56 (0.25-1.27)  0.17 | --- | 0.77 |
|  | Adj. for demographics | 0.91 (0.79-1.04)  0.16 | 0.85 (0.63-1.15)  0.30 | 0.54 (0.24-1.22)  0.14 | --- | 0.71 |
|  | Adj. for demographic and lifestyle factors | 0.91 (0.79-1.06)  0.23 | 0.83 (0.61-1.14)  0.26 | 0.52 (0.22-1.26)  0.15 | --- | 0.60 |
|  |  |  |  |  |  |  |
| Ever had premenopausal hot flashes | |  |  |  |  |  |
|  | Unadjusted | 0.99 (0.86-1.14)  0.87 | 0.80 (0.60-1.08)  0.14 | 2.60 (0.62-10.93)  0.19 | --- | 0.72 |
|  | Adj. for demographics | 1.00 (0.87-1.16)  1.00 | 0.82 (0.61-1.10)  0.19 | 2.49 (0.59-10.53)  0.21 | --- | 0.78 |
|  | Adj. for demographic and lifestyle factors | 1.02 (0.87-1.19)  0.85 | 0.85 (0.62-1.17)  0.32 | 2.00 (0.47-8.52)  0.35 | --- | 0.70 |
|  |  |  |  |  |  |  |
| Ever had postmenopausal hot flashes | |  |  |  |  |  |
|  | Unadjusted | 1.09 (0.96-1.25)  0.18 | 1.35 (1.01-1.81)  0.047 | 0.48 (0.20-1.19)  0.11 | --- | 0.77 |
|  | Adj. for demographics | 1.03 (0.90-1.19)  0.63 | 1.29 (0.95-1.74)  0.10 | 0.53 (0.21-1.33)  0.18 | --- | 0.67 |
|  | Adj. for demographic and lifestyle factors | 1.03 (0.89-1.19)  0.73 | 1.30 (0.94-1.79)  0.11 | 0.75 (0.28-1.98)  0.55 | --- | 0.38 |
|  |  |  |  |  |  |  |
| Ever had an oophorectomy | |  |  |  |  |  |
|  | Unadjusted | 1.04 (0.93-1.17)  0.47 | 1.12 (0.87-1.45)  0.38 | 1.01 (0.44-2.31)  0.98 | --- | 0.67 |
|  | Adj. for demographics | 1.03 (0.92-1.16)  0.60 | 1.16 (0.89-1.50)  0.27 | 1.03 (0.45-2.38)  0.95 | --- | 0.92 |
|  | Adj. for demographic and lifestyle factors | 1.01 (0.89-1.15)  0.90 | 1.14 (0.87-1.51)  0.34 | 1.08 (0.44-2.65)  0.86 | --- | 0.91 |
|  |  |  |  |  |  |  |
| Ever had a hysterectomy | |  |  |  |  |  |
|  | Unadjusted | 1.02 (0.91-1.13)  0.78 | 1.11 (0.87-1.40)  0.41 | 0.85 (0.40-1.80)  0.67 | --- | 0.48 |
|  | Adj. for demographics | 0.99 (0.88-1.10)  0.79 | 1.14 (0.90-1.45)  0.29 | 0.84 (0.39-1.80)  0.65 | --- | 0.82 |
|  | Adj. for demographic and lifestyle factors | 0.97 (0.86-1.10)  0.63 | 1.17 (0.90-1.51)  0.25 | 1.02 (0.45-2.32)  0.97 | --- | 0.67 |
| Results presented as odds ratio (95% confidence interval) and p-value. Demographic factors include age, race, ethnicity, region, and BMI. Lifestyle factors include smoking status, education, normalized socioeconomic status (NSES), and alcohol use. Policy from the Women’s Health Initiative will not allow researchers to report the number of participants in cells with fewer than 20 individuals. As such, cells that contain fewer than 20 participants read “<20”. | | | | | | |

| **Supplementary Table S10: Results from logistic regression analyses for the associations between preterm birth status and events related to menarche and menopause, stratified by age at enrollment** | | | | | |
| --- | --- | --- | --- | --- | --- |
|  | | **45-54 years OR (95% CI)** | **55-64 years OR (95% CI)** | **65-74 years OR (95% CI)** | **≥ 75 years OR (95% CI)** |
| N (preterm/term) | | 357/11,359 | 916/34,348 | 660/32,863 | 109/6,313 |
| Were periods regular | |  |  |  |  |
|  | Unadjusted | 0.79 (0.62-1.02) | 0.81 (0.69-0.95) | 0.77 (0.64-0.94) | 1.06 (0.61-1.83) |
|  | Adj for demographics | 0.90 (0.69-1.19) | 0.81 (0.69-0.95) | 0.76 (0.63-0.92) | 1.02 (0.59-1.77) |
|  | Adj for demographics and lifestyle factors | 0.85 (0.64-1.13) | 0.85 (0.71-1.01) | 0.74 (0.61-0.91) | 0.91 (0.52-1.59) |
|  |  |  |  |  |  |
| Ever had premenopausal hot flashes | |  |  |  |  |
|  | Unadjusted | 1.01 (0.76-1.34) | 0.86 (0.73-1.02) | 1.07 (0.86-1.33) | 0.70 (0.43-1.13) |
|  | Adj for demographics | 1.02 (0.76-1.37) | 0.86 (0.73-1.02) | 1.09 (0.87-1.35) | 0.66 (0.41-1.07) |
|  | Adj for demographics and lifestyle factors | 1.09 (0.79-1.52) | 0.88 (0.74-1.06) | 1.08 (0.85-1.36) | 0.73 (0.43-1.25) |
|  |  |  |  |  |  |
| Ever had postmenopausal hot flashes | |  |  |  |  |
|  | Unadjusted | 1.02 (0.77-1.37) | 1.04 (0.89-1.23) | 1.05 (0.88-1.27) | 1.33 (0.85-2.07) |
|  | Adj for demographics | 1.01 (0.75-1.36) | 1.02 (0.86-1.21) | 1.03 (0.86-1.25) | 1.35 (0.86-2.13) |
|  | Adj for demographics and lifestyle factors | 0.91 (0.66-1.26) | 1.05 (0.88-1.26) | 1.05 (0.86-1.28) | 1.32 (0.81-2.14) |
|  |  |  |  |  |  |
| Ever had an oophorectomy | |  |  |  |  |
|  | Unadjusted | 1.24 (0.99-1.55) | 1.13 (0.98-1.31) | 1.15 (0.98-1.36) | 1.06 (0.70-1.59) |
|  | Adj for demographics | 1.21 (0.96-1.53) | 1.13 (0.97-1.31) | 1.17 (0.99-1.38) | 1.03 (0.68-1.56) |
|  | Adj for demographics and lifestyle factors | 1.16 (0.89-1.50) | 1.07 (0.91-1.26) | 1.21 (1.01-1.44) | 0.97 (0.62-1.52) |
|  |  |  |  |  |  |
| Ever had a hysterectomy | |  |  |  |  |
|  | Unadjusted | 1.22 (0.99-1.51) | 1.07 (0.93-1.22) | 1.11 (0.95-1.30) | 0.92 (0.63-1.35) |
|  | Adj for demographics | 1.16 (0.93-1.45) | 1.05 (0.92-1.21) | 1.10 (0.94-1.29) | 0.90 (0.61-1.33) |
|  | Adj for demographics and lifestyle factors | 1.21 (0.95-1.54) | 1.02 (0.88-1.18) | 1.09 (0.92-1.29) | 0.92 (0.61-1.40) |
| Results presented as odds ratio (95% confidence interval). Demographic factors include age, race, ethnicity, region, and BMI. Lifestyle factors include smoking status, education, normalized socioeconomic status (NSES), and alcohol use. | | | | | |

| **Supplementary Table S11: Results from logistic regression analyses for the associations between preterm birth status and events related to menarche and menopause, stratified by self-reported race and ethnicity** | | | | | | | |
| --- | --- | --- | --- | --- | --- | --- | --- |
|  | | **Race** | | | | **Ethnicity** | |
|  |  | **Asian OR (95% CI)** | **Black OR (95% CI)** | **White OR (95% CI)** | **Other OR (95% CI)** | **Hispanic/Latinx OR (95% CI)** | **Not Hispanic/Latinx OR (95% CI)** |
| N (preterm/term) | | 44/2,327 | 162/6,621 | 1,770/72,954 | 26/1,135 | 83/3,588 | 1,941/80,472 |
| Were periods regular | |  |  |  |  |  |  |
|  | Unadjusted | 0.55 (0.28-1.08) | 0.60 (0.40-0.88) | 0.83 (0.73-0.93) | 0.65 (0.27-1.58) | 0.91 (0.52-1.60) | 0.79 (0.71-0.88) |
|  | Adj for demographics | 0.57 (0.27-1.20) | 0.60 (0.40-0.90) | 0.84 (0.75-0.95) | 0.84 (0.30-2.36) | 1.17 (0.54-2.53) | 0.81 (0.72-0.91) |
|  | Adj for demographics and lifestyle factors | 0.53 (0.24-1.18) | 0.54 (0.35-0.84) | 0.85 (0.75-0.96) | 0.90 (0.28-2.86) | 1.54 (0.64-3.70) | 0.80 (0.71-0.91) |
|  |  |  |  |  |  |  |  |
| Ever had premenopausal hot flashes | |  |  |  |  |  |  |
|  | Unadjusted | 1.23 (0.51-2.92) | 1.02 (0.69-1.51) | 0.91 (0.80-1.03) | 0.63 (0.26-1.51) | 1.46 (0.77-2.77) | 0.91 (0.81-1.02) |
|  | Adj for demographics | 1.08 (0.44-2.61) | 1.08 (0.72-1.62) | 0.93 (0.82-1.05) | 0.72 (0.26-2.02) | 1.44 (0.67-3.10) | 0.93 (0.82-1.04) |
|  | Adj for demographics and lifestyle factors | 0.91 (0.37-2.24) | 0.99 (0.63-1.54) | 0.96 (0.84-1.10) | 0.81 (0.26-2.52) | 2.22 (0.87-5.71) | 0.94 (0.83-1.07) |
|  |  |  |  |  |  |  |  |
| Ever had postmenopausal hot flashes | |  |  |  |  |  |  |
|  | Unadjusted | 0.98 (0.48-2.02) | 1.17 (0.77-1.77) | 1.09 (0.97-1.23) | 1.34 (0.53-3.36) | 1.11 (0.65-1.88) | 1.10 (0.98-1.23) |
|  | Adj for demographics | 0.96 (0.45-2.09) | 1.09 (0.71- 1.67) | 1.04 (0.92-1.17) | 1.32 (0.46-3.82) | 0.83 (0.41-1.65) | 1.05 (0.94-1.18) |
|  | Adj for demographics and lifestyle factors | 0.92 (0.40-2.08) | 1.23 (0.74-2.03) | 1.04 (0.92-1.18) | 1.06 (0.34-3.30) | 0.67 (0.32-1.42) | 1.06 (0.94-1.20) |
|  |  |  |  |  |  |  |  |
| Ever had an oophorectomy | |  |  |  |  |  |  |
|  | Unadjusted | 1.83 (0.98-3.42) | 0.83 (0.60-1.16) | 1.15 (1.04-1.28) | 0.96 (0.41-2.24) | 1.48 (0.93-2.36) | 1.13 (1.03-1.24) |
|  | Adj for demographics | 1.98 (1.02-3.82) | 0.87 (0.62-1.22) | 1.16 (1.05-1.29) | 1.36 (0.54-3.39) | 1.37 (0.76-2.49) | 1.15 (1.04-1.26) |
|  | Adj for demographics and lifestyle factors | 1.55 (0.75.3.23) | 0.76 (0.51-1.12) | 1.15 (1.03-1.28) | 1.72 (0.66-4.48) | 1.10 (0.57-2.13) | 1.13 (1.01-1.25) |
|  |  |  |  |  |  |  |  |
| Ever had a hysterectomy | |  |  |  |  |  |  |
|  | Unadjusted | 1.57 (0.86-2.86) | 0.91 (0.66-1.24) | 1.09 (0.99-1.20) | 0.81 (0.37-1.78) | 1.53 (0.99-2.37) | 1.08 (0.98-1.18) |
|  | Adj for demographics | 1.76 (0.91-3.38) | 0.92 (0.67-1.27) | 1.08 (0.98-1.19) | 1.10 (0.46-2.65) | 1.26 (0.72-2.19) | 1.08 (0.98-1.18) |
|  | Adj for demographics and lifestyle factors | 1.25 (0.60-2.60) | 0.92 (0.64-1.33) | 1.07 (0.97-1.19) | 1.36 (0.54-3.44) | 1.00 (0.55-1.83) | 1.07 (0.97-1.18) |
| Results presented as odds ratio (95% confidence interval). Demographic factors include age, race, ethnicity, region, and BMI. Lifestyle factors include smoking status, education, normalized socioeconomic status (NSES), and alcohol use. | | | | | | | |
